# Can acupuncture reverse oxidative stress and neuroinflammatory damage in animal models of vascular dementia?: A preclinical systematic review and meta-analysis

**DOI:** 10.1097/MD.0000000000033989

**Published:** 2023-06-09

**Authors:** Qin Wen, Xueqin Hong, Kunze He, Buping Liu, Min Li

**Affiliations:** a Clinical Medical College of Acupuncture Moxibustion and Rehabilitation, Guangzhou University of Chinese Medicine, Guangzhou, China; b Yuangang Street Community Health Service Center, Guangzhou, China.

**Keywords:** acupuncture, animal models, preclinical meta-analysis, preclinical systematic review, vascular dementia

## Abstract

Vascular dementia is a cognitive dysfunction syndrome caused by cerebral vascular factors such as ischemic stroke and hemorrhagic stroke. The effect of acupuncture on vascular dementia models is ambiguous, and there is controversy about whether acupuncture has a placebo effect. Oxidative stress and inflammation are the most essential mechanisms in preclinical studies of vascular dementia. However, there is no meta-analysis on the mechanism of vascular dementia in animal models. It is necessary to explore the efficacy of acupuncture through Meta-analysis of preclinical studies.

**Methods:**

Three major databases, PubMed, Embase and Web of Science (including medline), were searched in English until December 2022.The quality of the including literature was assessed using SYRCLE’s risk of bias tool. Review Manager 5.3 was used to statistically summarize the included studies and the statistical effect values were expressed by SMD. The outcomes included: behavioral tests (escape latency, number of crossings), pathological sections (Nissl and TUNEL staining), oxidative stress markers (ROS, MDA, SOD, GSH-PX) and neuroinflammatory factors (TNF-α, IL-1β, IL-6).

**Results:**

A total of 31 articles were included in this meta-analysis. The results showed that the escape latency, the contents of ROS, MDA, IL-1β, and IL-6 were decreased, and the contents of SOD and Nissl-positive neurons were increased in the acupuncture group as compared with the non-group (*P* < .05). Compared with the impaired group, the acupuncture group also had the above advantages (*P* < .05). In addition, the acupuncture group also increased the number of crossings and GSH-PX content, and decreased the expression of TUNEL-positive neurons and TNF-α (*P* < .05).

**Conclusions:**

From behavioral tests to slices and pathological markers in animal models of vascular dementia, it can be proved that acupuncture is effective in targeting oxidative stress and neuroinflammatory damage, and acupuncture is not a placebo effect. Nevertheless, attention needs to be paid to the gap between animal experiments and clinical applications.

## 1. Introduction

Human cognition is complex and multidimensional in daily life, and recovery after damage is difficult.^[1]^ Vascular dementia (VD) is a group of conditions dominated by cognitive dysfunction that occurs in more than 33% of patients with cerebral hypoperfusion disorders.^[2]^ However, most of these patients are inadequately treated.^[3]^ Although the U.S. Food and Drug Administration offers effective ways to improve cognitive function, these drugs are not specifically targeted for patients with VD, and the side effects of drug interventions create difficulties in the therapy of VD patients.^[4,5]^ Therefore, a multifaceted and holistic approach is necessary to improve and complement the existing treatments.

The ongoing global population aging crisis has exacerbated the challenge of managing people with dementia. Although some countries have introduced policies related to the management of dementia patients, many patients do not receive good care due to uneven regional development.^[6,7]^ As the financial burden on governments increases, government organizations are calling for the use of non-pharmaceuticals as an important intervention for dementia.^[8,9]^ Recently, several authoritative journals have published articles indicating that acupuncture has a significant impact on neurological diseases, with VD being one of the major diseases treated with acupuncture.^[10,11]^ A Delphi expert consensus survey for cognitive impairment showed that more than 80% of the experts who participated in the survey agreed that acupuncture can relive mild or moderate cognitive impairment caused by cerebrovascular diseases.^[12]^

With the continuous progress of biomedicine, acupuncture intervention in VD animal models has attracted more and more attention, mainly because they provide more accurate results for clinical research and provide insight into the intrinsic mechanisms of the therapy. During acupuncture treatment for neurological disorders, oxidative stress, inflammation, and apoptosis decrease, and neurotransmitter levels and synaptic plasticity increase.^[13]^ A major pathological mechanism of VD is oxidative stress, which triggers inflammation and induces neuronal death.^[14]^ chronic cerebral hypoperfusion is the main cause of VD and a key step of oxidative stress. After oxidative stress, mitochondrial dysfunction occurs and Reactive Oxygen Species (ROS) and reactive nitrogen species are generated.^[15]^ The original redox homeostasis in the body is broken, resulting in the destruction of cellular proteins, lipids, sugars and DeoxyriboNucleic Acid (DNA). When oxidative stress is uncontrolled, the number of damaged neurons accumulates continuously. Microglia cells in the brain are activated under the stimulation of lipid peroxides, releasing excessive inflammatory factors and inducing neuroinflammatory response. Studies have shown that the double crosstalk of oxidative stress and inflammation is the prelude to apoptosis. Under the double pathological factors, the caspase family and the pro-apoptotic factors in the Bcl-2 family jointly perform neuronal cell apoptosis, and finally lead to cognitive dysfunction.^[16]^ According to recent studies, it also plays a role in ferroptosis and cuproptosis, 2 newly described cell death processes.^[17,18]^ Above, many studies focus on the effect of acupuncture on oxidative stress in VD animal models.

However, those who did animal studies of acupuncture for VD emphasized different outcomes, and the same results showed different effects.^[13]^ As a result, inconsistent evidence has increased uncertainty about the efficacy of acupuncture. To clarify the efficacy of acupuncture and enhance the strength of the evidence, we performed a meta-analysis. As far as we know, this is the first meta-analysis to incorporate behavioral tests, sections, and cytokines. The following hypotheses were tested: acupuncture is more effective in improving cognition in animal models of VD than is untreated palliative care and non-acupoints, and In terms of mechanisms related to oxidative stress, inflammation, and apoptosis, acupuncture has advantages.

## 2. Materials and methods

### 2.1. Search strategy

According to PRISMA 2020 guideline, the meta-analysis was conducted.^[19]^ The INPLASY registration number for this study was INPLASY202330114, and there was no previous registration for the same study. Our article is meta-analysis, based on existing published literature and did not require ethical approval. The databases of PubMed, Embase, and Web of Science (including Medline) were searched independently by 2 authors in English. Search database from inception to December 2022. Each term, either alone or in combination, was searched for: “acupuncture,” “acupoint,” “electroacupuncture,” “vascular dementia,” “vascular cognitive impairment,” and “infarct dementia.”

### 2.2. Inclusion and exclusion criteria

Following is a list of PICO criteria that were applied to all included studies.

#### 2.2.1. Inclusion criteria.

Participants: Various surgical methods were used to modeling VD animals. Breeds, genders, weights, and ages of the animals were not restricted.

Intervention: Surgical modeling was followed by acupuncture treatment without restrictions on acupuncture prescription (scheme, intensity, duration of treatment).

Comparison: Animal models of VD without any intervention (impaired group) and those receiving shame acupuncture treatment (non-acupoint group).

Outcomes: Morris water maze test results (escape latency, number of original platform crossings), number of Nissl-positive neurons and proportion of terminal deoxynucleotidyl transferase dUTP nick end labeling (TUNEL)-positive neurons, superoxide dismutase (SOD) (U/mg · protein) and glutathione peroxidase (GSH-PX) (U/mg · protein) activity, ROS level, malondialdehyde amount (MDA) (nmol/mg · protein), tumor necrosis factor-α (TNF-α) (pg/mL), interleukin-1β (IL-1β) (pg/mL), and interleukin-6 (IL-6) (pg/mL) levels.

#### 2.2.2. Exclusion criteria.

1. Research on acupuncture combined with other therapies.
2. Non-VD research.
3. Experiments with animals that do not use randomized control methods.
4. VD animal study without oxidative stress, inflammation, apoptosis mechanisms.
5. Reviews, abstracts, conference papers, and dissertations.
6. Studies using different units of measure for each same outcome (not interchangeable).
7. Repeated and data-identical studies.

### 2.3. Data extraction

Data were extracted by 2 independent researchers, then another author reconcile the data. Extracted data included the following (Tables 1 and 2): author information, basic animal information, anesthetic, surgical operation, treatment group and sample size, control group and sample size, outcomes; acupuncture mode, acupuncture points, stimulation intensity, time per treatment, and total treatment duration.

**Table 1:** Basic information of literature.

Study (years)	Species (sex)	Age	Weight (g)	Anesthetic	Model method	Treatment group (sample size)	Control group (sample size)		Outcomes	Mechanism
Qiu et al, 2022	SD rats ——	——	220–250	Chloral hydrate (30 mg/100 g)	2VO	EA (6)	2VO (10)	——	①Escape latency ②Number of crossings ⑪ROS	Oxidative stress Apoptosis Inflamtion
Li et al, 2021	Wistar rats (male)	4–5 mo	300–320	Chloral hydrate (10 mL/kg)	2VO	MA (18)	2VO (18)	Non-Acu (18)	①Escape latency ⑤SOD ⑥MDA	Oxidative stress
Su et al, 2019	SD rats (male)	2 mo	220 ± 20	100 mg/kg ketamine plus 10 mg/kg xylazine	MACO	MA (10)	MCAO (10)	——	①Escape latency ②Number of crossings ④TUNEL-positive cell ratio	Oxidative stress Apoptosis Inflamtion
Yang et al, 2018	Wistar rats (male)	8 wk	280–320	pentobarbital sodium (50 mg/kg)	2VO	MA (6)	2VO (6)	Non-Acu (6)	①Escape latency ③Nissl-positive neurons ⑪ROS	Oxidative stress
Du SQ et al, 2018	Wistar rats (male)	10 wk	——	Pentobarbital sodium (40 mg/kg)	2VO	MA (10)	2VO (10)	——	①Escape latency ③Nissl-positive neurons ⑤SOD	Oxidative stress Inflamtion Apoptosis
Li et al, 2016	Wistar rats (male)	Adult	270–320	Chloral hydrate (35 mg/100 g)	2VO	MA (8)	2VO (8)	Non-Acu (8)	①Escape latency	Oxidative stress
Wang et al, 2015	Wistar rats (male)	——	200–220	pentobarbital sodium (40 mg/kg)	2VO	MA (10)	2VO (10)	Non-Acu (10)	①Escape latency ⑪ROS	Oxidative stress Inflamtion
Lin et al, 2015	SD rats (male)	Adult	250–280	10% chloral hydrate (300 mg/kg)	MACO	EA (24)	MCAO (24)	——	⑤SOD ⑥MDA ⑦GSH-PX	Oxidative stress Apoptosis
Zhang et al, 2014	Wistar rats (male)	——	300–320	——	EO	MA (10)	EO (10)	Non-Acu (10)	①Escape latency ②Number of crossings ⑤SOD ⑥MDA	Oxidative stress
Zhu et al, 2013	SD rats (female)	12 mo	432 ± 30	10% chloral hydrate (3.5 mg/kg)	2VO	EA (6)	2VO (6)	——	①Escape latency	Oxidative stress
He et al, 2012	Wistar (male)	Adult	200 ~ 220 g	——	4VO	4VO (12)	ACU (9)	——	⑤SOD	Oxidative stress
Yang et al, 2007	Wistar rats ——	Adult	200–250	Pentobarbital sodium (40 mg/kg)	2VO	MA (6)	2VO (6)	——	⑤SOD ⑥MDA	Oxidative stress
Liu et al, 2006	Wistar rats (male)	Adult	340 ± 40	6.5% chloral hydrate (35 mg/100 g)	EO	MA (15)	EO (14)	Non-Acu (14)	②Number of crossings ⑤SOD ⑦GSH-PX	Oxidative stress
Wang et al, 2004	SD rats (male/female)	2–3 mo	200–250	6.5% chloral hydrate (0.3 mL/100 g)	4VO	EA (14)	4VO (13)	——	①Escape latency ②Number of crossings ⑤SOD ⑦GSH-PX	Oxidative stress
Ma et al, 2022	SD rats (male)	8 wk	200–280	100 mg/kg of ketamine and 10 mg/kg xylazine	2VO	EA (12)	2VO (12)	Non-Acu (12)	②Number of crossings ④TUNEL-positive cell ratio	Apoptosis
Wang et al, 2021	SD rats (male)	10–12 wk	260 ± 20	——	MACO	EA (9)	MCAO (9)	——	①Escape latency ②Number of crossings	Apoptosis
Guo et al, 2020	SD rats (male)	——	200 ± 20	Chloral hydrate (350 mg/kg)	2VO	EA (10)	2VO (10)	——	①Escape latency ②Number of crossings ③Nissl-positive neurons ④TUNEL-positive cell ratio	Apoptosis
Zhu et al, 2018	Wistar rats (male)	——	200–220	Pentobarbital sodium (40 mg/kg)	2VO	MA (10)	2VO (10)	Non-Acu (10)	①Escape latency ⑪ROS	Oxidative stress Apoptosis
Tian et al, 2015	SD rats (male)	——	280 ± 20	chloral hydrate (3.5 mL/kg)	4VO	MA (8)	4VO (8)	——	①Escape latency ②Number of crossings	Apoptosis
Feng et al, 2013	SD rats (male)	——	250–280	10% chloral hydrate	MACO	EA (15)	MCAO (15)	——	①Escape latency ②Number of crossings ④TUNEL-positive cell ratio	Apoptosis
Wang et al, 2009	Wistar (male)	10 mo	300 ± 40	6.5% chloral hydrate (35 mg/100 g)	EO	MA (12)	EO (11)	Non-Acu (11)	④TUNEL-positive cell ratio	Apoptosis
Xu et al, 2022	Wistar rats (male)	8 wk	280 ± 20	Pentobarbital sodium (50 mg/kg)	2VO	MA (12)	2VO (12)	——	①Escape latency ②Number of crossings	Inflamtion
Chen et al, 2022	SD rats _	——	220–250	Chloral hydrate (30 mg/100 g)	2VO	EA (10)	2VO (10)	——	①Escape latency ⑨IL-1β	Inflamtion
Bu et al, 2022	SD rats (male)	——	180–200	Pentobarbital sodium (40 mg/kg)	4VO	EA (6)	4VO (6)	——	①Escape latency ②Number of crossings ⑧TNF-α ⑩IL-6	Inflamtion
Pan et al, 2021	Wistar rats (male)	2 mo	260–280	Pentobarbital sodium (40 mg/kg)	2VO	MA (10)	2VO (10)	Non-Acu (10)	①Escape latency ②Number of crossings ⑧TNF-α ⑨IL-1β	Inflamtion
Cao et al, 2021	Wistar rats (male)	7–8 wk	260–300	Pentobarbital sodium (40 mg/kg)	2VO	MA (5)	2VO (5)	——	①Escape latency ③Nissl-positive neurons ⑧TNF-α ⑩IL-6	Inflamtion
Ma et al, 2020	Wistar rats (male)	——	260–280	Pentobarbital sodium (40 mg/kg)	2VO	MA (8)	2VO (8)	Non-Acu (8)	①Escape latency ②Number of crossings ⑧TNF-α ⑨IL-1β ⑩IL-6	Inflamtion
Wang et al, 2020	Wistar rats (male)	——	270–320	Pentobarbital sodium (40 mg/kg)	2VO	MA (6)	2VO (6)	——	①Escape latency ⑧TNF-α ⑩IL-6	Inflamtion
Liu et al, 2017	SD rats (male)	10–12 wk	250–280	10% chloral hydrate (3ml/kg)	MACO	EA (9)	MCAO (8)	——	①Escape latency ②Number of crossings	Inflamtion
Han et al, 2017	SD rats (male)	3–6 mo	200–220	10% chloral hydrate (300 mg/kg)	2VO	EA (10)	2VO (10)	——	①Escape latency ⑧TNF-α ⑨IL-1β	Inflamtion
Li et al, 2007	SD rats (male/female)	2–3 mo	200–250	——	4VO	EA (12)	4VO (11)	——	①Escape latency ②Number of crossings	Inflamtion

2VO = bilateral common carotid artery occlusion, 4VO = 4-vessel occlusion, EA = electroacupuncture, EO = embolic occlusion, GSH-PX = glutathione peroxidase activity, IL-1β = interleukin-1β, IL-6 levels = interleukin-6, MA = manual acupuncture, MCAO = middle cerebral artery occlusion, MDA = malondialdehyde amount, Non-Acu = non-acupoint, ROS = reactive oxygen species, SD rats = Sprague-Dawley rats, SOD = superoxide dismutase activity, TNF-α = tumor necrosis factor-α.

**Table 2:** Acupuncture information.

Study (years)	Acupuncture	Acupoints	Stimulus intensity	Time	Course (d)
Li et al, 2021	MA	Baihui (GV20), bilateral Zusanli (ST36)	——	——	14
Cao et al, 2021	MA	Baihui (GV20), bilateral Zusanli (ST36)	——	10 min	14
Ma et al, 2020	MA	Baihui (GV20), bilateral Zusanli (ST36)	30 s, Twisting angle < 90, Frequency > 120	30 s	14
Wang et al, 2020	MA	Baihui (GV20), bilateral Zusanli (ST36)	——	10 min	14
Yang et al, 2018	MA	Baihui (GV20), bilateral Zusanli (ST36)	30 s, Twisting angle < 90, Frequency > 120	30 s	14
Zhu et al, 2018	MA	Baihui (GV20), bilateral Zusanli (ST36)	——	10 min	14
Du et al, 2018	MA	Baihui (GV20), bilateral Zusanli (ST36)	Twice a second for 30 s	30 s	14
Li et al, 2016	MA	Baihui (GV20), bilateral Zusanli (ST36)	Twice a second for 30 s	30 s	14
Wang et al, 2015	MA	Baihui (GV20), bilateral Zusanli (ST36)	——	1 min	14
Xu et al, 2022	MA	Danzhong (CV17), Qihai (CV6), Zhongwan (CV12), bilateral Zusanli (ST36), bilateral Xuehai (SP10)	Twisting method 30 s	30 s	15
Pan et al, 2021	MA	Danzhong (CV17), Qihai (CV6), Zhongwan (CV12), bilateral Zusanli (ST36), bilateral Xuehai (SP10)	——	30 s	14
Zhang et al, 2014	MA	Danzhong (CV17), Qihai (CV6), Zhongwan (CV12), bilateral Zusanli (ST36), bilateral Xuehai (SP10)	——	30 s	21
Liu et al, 2006	MA	Danzhong (CV17), Qihai (CV6), Zhongwan (CV12), bilateral Zusanli (ST36), bilateral Xuehai (SP10)	Twice a second for 30 s	30 s	21
Wang et al, 2009	MA	Danzhong (CV17), Qihai (CV6), Zhongwan (CV12), bilateral Zusanli (ST36), bilateral Xuehai (SP10)	Twice a second for 30 s	30 s	21
Qiu et al, 2022	EA	Baihi (GV20), Dazhui (GV14), Shenshu (BL23)	Disperse-dense wave, 10/50 Hz, 1.0 mA	30 min	28
Chen et al, 2022	EA	Baihi (GV20), Dazhui (GV14), Shenshu (BL23)	Disperse-dense wave,10 Hz/50 Hz	30 min	28
Zhu et al, 2013	EA	Baihi (GV20), Dazhui (GV14), Shenshu (BL23)	Continuous wave, 4 Hz, 2.0 mA	20 min	30
Han et al, 2017	EA	Baihui (GV20), Dazhui (GV14)	Dilatational wave, 2/15 Hz, 2 ± 1 mA	30 min	28
Li et al, 2007	EA	Baihui (GV20), Dazhui (GV14)	Continuous wave, 50 Hz, 2.0 mA	20 min	15
Wang et al, 2004	EA	Baihui (GV20), Dazhui (GV14)	Continuous wave, 150 Hz, 2.0 mA	20 min	15
Wang et al, 2021	EA	Baihui(GV20), Shenting (GV24)	Dilatational wave, 1–20 Hz, 2 mA	30 min	8
Ma et al, 2022	EA	Baihui (GV20), Shenting (GV24)	Longitudinal wave, 2 Hz, 3.0 mA	20 min	14
Liu et al, 2017	EA	Baihui (GV20), Shenting (GV24)	Dispersed, 2–10 Hz, 2-4 mA	30 min	7
Lin et al, 2015	EA	Baihui (GV20), Shenting (GV24)	Dispersed, 5–20 Hz, 1-3 mA	30 min	7
Feng et al, 2013	EA	Baihui (GV20), Shenting (GV24)	Disperse waves, 1–20 Hz	30 min	10
Bu et al, 2022	EA	Baihui (DV20), Danzhong (CV17), Geshu (BL17), Qihai (CV6), Sanyinjiao (SP6)	Sparse-dense wave, 2/15 Hz, 1 mA	30 min	21
Guo et al, 2020	EA	Baihui (GV20), Dazhui (GV14), Geshu (BL17), Housanli	Sparse wave, 2 Hz, 2 mA	10 min	14
Su et al, 2019	MA	Baihui (GV20), Dazhui (GV14), Renzhong (GV26), Fengfu (GV16)	——	20 min	15
Tian et al, 2015	MA	Daomoshangjiao, Jiyi, Siwei	Twisting method 30 s	30 min	10
He et al, 2012	EA	Shaochong (HT9), Zhongchong (PC 9), Yongquan (K11), Shaoshang (LU 11)	Sparse-dense wave, 2–100 Hz, 1–3 mA	20 min	14
Yang et al, 2007	MA	Baihui (GV20), Dazhui (GV14), Shuigou (GV26)	Continuous twist repeated many times	1 min	15

EA = electroacupuncture, MA = manual acupuncture.

Some of the included studies involved multiple intervention groups, and we only extracted data from the acupuncture, impaired, and non-acupoint groups. The outcomes of this study were continuous variables, and the mean, standard deviation (SD), and number of participants were extracted. If standard error (SE) appeared in the text, we used a formula to convert SE to SD and then performed a summary analysis. When acupuncture groups with different treatment frequencies emerged in the article, data from the highest frequency group were extracted. If the included literature has no exact data and only provides graphical results, we try to reach out to the authors to obtain raw data or scanned it with software (GetData Graph Digitizr 2.26).^[20]^

### 2.4. Risk of bias

The Systematic Review Center for Laboratory Animal Experimentation (SYRCLE)’s risk of bias tool was used by 2 independent investigators to assess each included study for bias.^[21]^ There were disagreements among the authors, which were resolved by discussion with a third author.

### 2.5. Statistical analysis

Data analysis was carried out using Review Manager 5.3 (Cochrane Collaboration, Copenhagen, Denmark). Continuous variables were presented as standard mean difference (SMD) with 95% confidence interval (CI). The first step in the analysis was to determine if the studies were homogeneous or heterogeneous. The fixed-effects (FE) model was used for the analysis when the studies were homogeneous (*P* ≥ .05, *I*^2^ ≤ 50%), In cases where the studies were heterogeneity (*P* < .05, *I*^2^ > 50%), we employed an random-effects (RE) model. Sensitivity analyses were then performed to assess the stability of the results and to identify sources of heterogeneity. Lastly, we analyzed possible publication bias using a funnel plot.

#### 2.5.1. Subgroup analysis.

To evaluate the differences between the impaired and non-acupoint groups, we performed a group analysis of some results. The effects of different acupuncture protocols in the Morris water maze trial were observed in terms of acupuncture point combinations, methods and total treatment duration groupings. In addition, we did a subgroup analysis of the efficacy of acupuncture in different surgical models.

## 3. Results

### 3.1. Screening process

**Figure 1.**
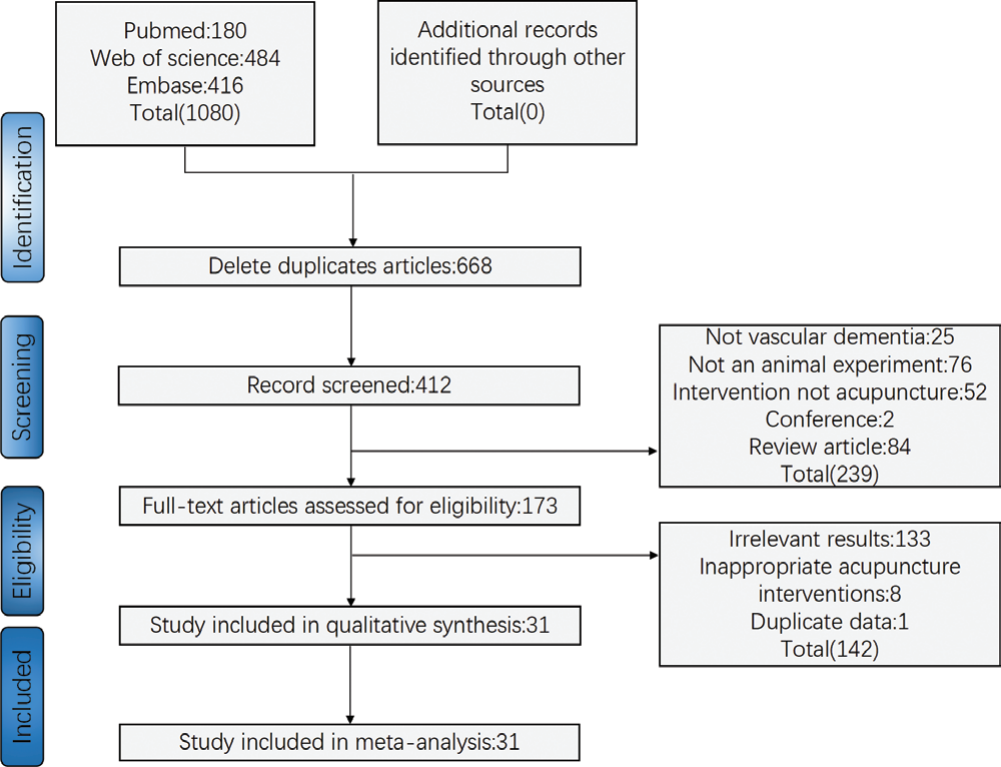
Screening process.

Two researchers retrieved 1080 publications from PubMed, Embase, Medline, and Web of Science. In the first step, 668 duplicates were removed, followed by an analysis of the titles and abstracts to eliminate 239 documents, including acupuncture combined with other therapy researches, non-VD studies, non-randomized control animal studies, and review papers; Then, 142 documents with non-targeted outcomes and similar information were excluded after reading the full text; and finally, 31 documents that met the inclusion criteria were analyzed (Fig. 1).

### 3.2. Characteristics of included studies in the meta-analysis

This meta-analysis included 31 articles (Table 1), including 23^[22–44]^in English and 8^[45–52]^ in Chinese. All articles were published between June 2004 and May 2022. All studies were carried out in China, with a total of 751 animals. Studies that included the Baihui (GV20) + bilateral Zusanli (ST36)^[29,30,32,38,39,41–43,50]^ and Qihai (CV6) + Zhongwan (CV12) + Danzhong (CV17) + bilateral Xuehai (SP10) + bilateral Zusanli (ST36)^[26,44,46]^ combinations of acupoints used manual acupuncture, and studies that included Baihui (GV20) + Dazhui (GV14),^[36,40,47]^ Baihui (GV20) + Dazhui (GV14) + bilateral Shenshu (BL23),^[37,45,51]^ and Baihui (GV20) + Shenting (GV24)^[25,28,33]^ all used electroacupuncture.

The stimulation of manual acupuncture was mainly based on the twisting of the acupuncture needles. The time for a single acupuncture session was mainly 30 seconds or 10 minutes, and the course of treatment was mainly 14 or 21 days. The stimulation of electroacupuncture was mainly continuous or density wave, the intensity was 2 to 50 Hz, the current was 1 to 3 mA, each treatment lasted 20 to 30 minutes, and the course of treatment was approximately 1 week, half a month, or 1 month (Table 2).

### 3.3. Quality evaluation

**Figure 2.**
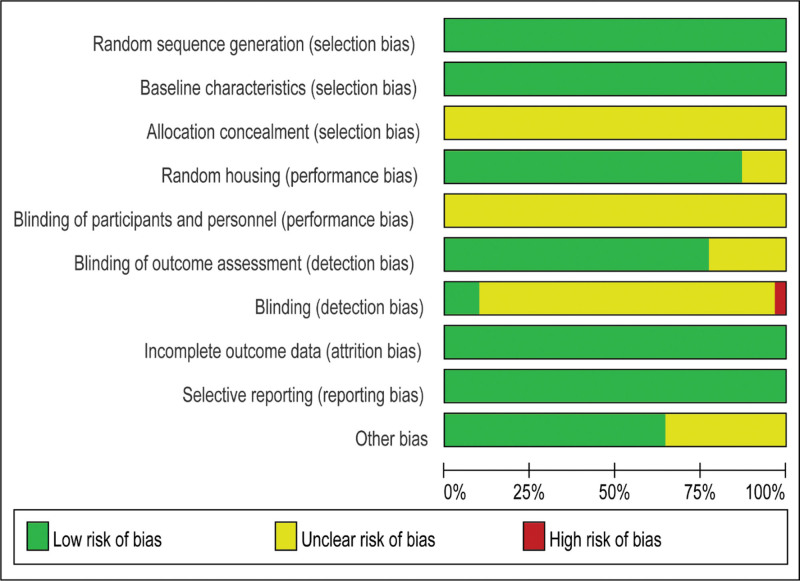
Risk of bias graph.

In general, thirteen studies were of high quality,^[23,25,29,32,34,35,41,43,45,46,48,49,51]^ and the rest were of moderate quality^[22,24,26–28,30,31,33,36–40,42,44,47,50,52]^ (Fig. 2). All studies mentioned random assignment, and 4 explicitly described randomization methods.^[32,47,49,51]^ All papers were baseline-balanced, but none mentioned allocation concealment schemes. Twenty-seven studies explained the breeding environment, and 4 studies did not.^[31,36,47,52]^ Because the intervention was acupuncture, the operator could not use blinding during the procedure. Six articles did not specify whether the animals they assessed were randomly selected.^[22,26–28,39,40]^ In terms of outcome statistics, 3 article explicitly mentioned blinding,^[40–42]^ 1 article was completed by the first author,^[37]^ and statistical blinding was not mentioned in the remaining 27 articles. Results from all studies were complete and no selective reporting was assessed. In terms of other biases^[22,23,25,27–29,32,34–37,39–41,45,46,48,49,51,52]^ had no other biases, and the remaining 11 articles^[24,26,30,31,33,38,42–44,47,50]^ did not mention the amount of acupuncture stimulation or use of anesthetics (Figure S1, Supplemental Digital Content, http://links.lww.com/MD/J110).

### 3.4. Meta-analysis

#### 3.4.1. Escape latency.

**Figure 3.**
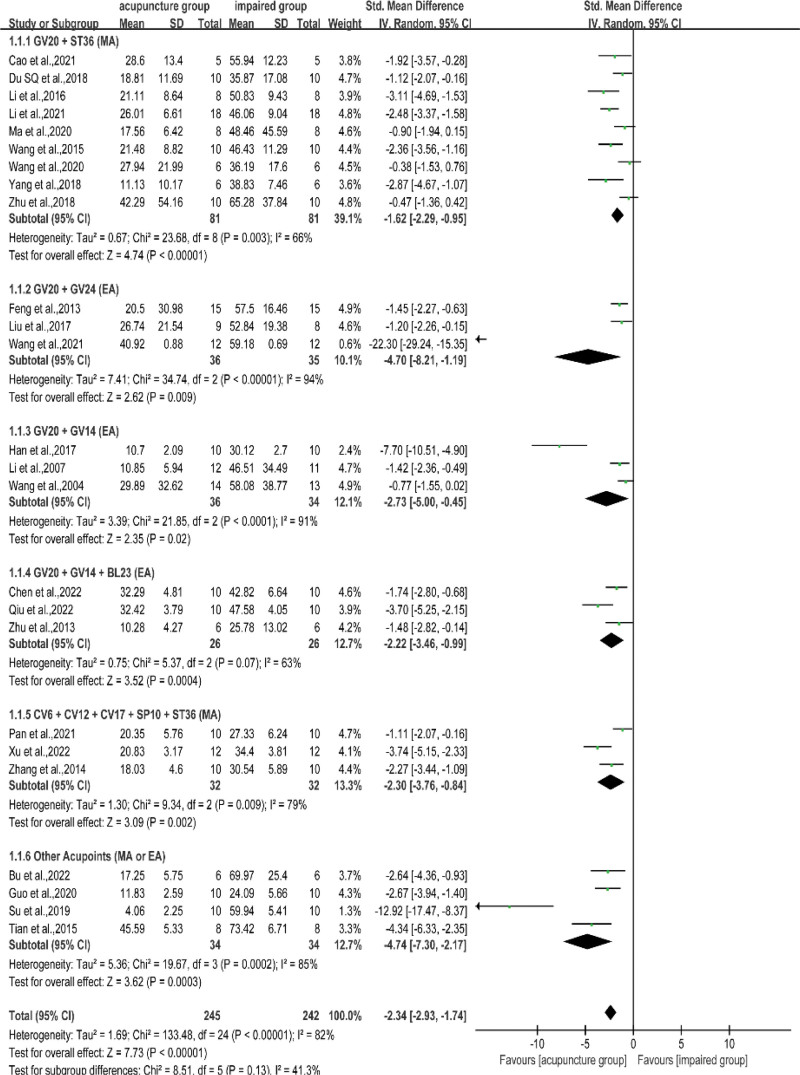
Subgroup analysis of escape latency between the acupuncture and the impaired groups. Baihui (GV20) + bilateral Zusanli (ST36), Baihui (GV20) + Shenting (GV24), Baihui (GV20) + Dazhui (GV14), Baihui (GV20) + Dazhui (GV14) + bilateral Shenshu (BL23), Qihai (CV6) + Zhongwan (CV12) + Danzhong (CV17) + bilateral Xuehai (SP10) + bilateral Zusanli (ST36), Other Acupoints (Acupuncture points and methods are different for each study). CI = confidence interval, EA = electroacupuncture, MA = manual acupuncture, SD = standard deviation.

**Figure 4.**
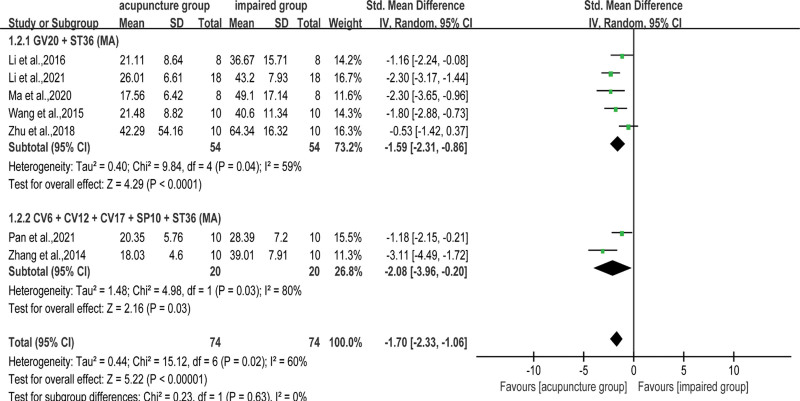
Subgroup analysis of escape latency between the acupuncture and the non-acupoint groups. Baihui (GV20) + bilateral Zusanli (ST36), Qihai (CV6) + Zhongwan (CV12) + Danzhong (CV17) + bilateral Xuehai (SP10) + bilateral Zusanli (ST36). CI = confidence interval, MA = manual acupuncture, SD = standard deviation.

Evasion latency is the main behavioral outcome to evaluate the efficacy of acupuncture in alleviating cognitive dysfunction in VD rats. Information about escape latency between the acupuncture and impaired groups was available in 25 studies. The pooled SMD applied in an RE model revealed a shortened escape latency for VD rats treated with acupuncture (SMD = −2.34, 95% CI: −2.93 to −1.74, *I*^2^ = 82%, *P* < .001, Fig. 3). According to the pooled results of 7 articles, the escape latency of the acupuncture group in the RE model was shorter than that of the non-acupoint group (SMD = −1.70, 95% CI: −2.33 to −1.06, *I*^2^ = 60%, *P* < .001, Fig. 4). Considering the results showed heterogeneity, different prescriptions (Table 2) were examined in subgroup analyses.

When comparing acupuncture and impaired groups, acupuncture treatment delivered to VD rats as GV20 + ST36^[29,30,32,38,39,41–43,50]^ (SMD = −1.62, 95% CI: −2.29 to −0.95, *I*^2^ = 66%, *P* < .001), GV20 + GV24^[25,28,33]^ (SMD = −4.70, 95% CI: −8.21 to −1.19, *I*^2^ = 94%, *P* < .001), GV20 + GV14^[36,40,47]^ (SMD = −2.73, 95% CI: −5.00 to −0.45, *I*^2^ = 91%, *P* = .02), GV20 + GV14 + BL23^[37,45,51]^ (SMD = −2.22, 95% CI: −3.46 to −0.99, *I*^2^ = 63%, *P* < .001), CV6 + CV12 + CV17 + SP10 + ST36^[26,44,46]^ (SMD = −2.30, 95% CI: −3.76 to −0.84, *I*^2^ = 79%, *P* = .002), or other acupoints^[31,34,48,49]^ (SMD = −4.74, 95% CI: −7.30 to −2.17, *I*^2^ = 85%, *P* < .001) had a similar effect on shortened escape latency. The subgroup analysis indicated that, except for GV20 + ST36 and GV20 + GV14 + BL23 prescriptions, the intragroup heterogeneity of the remaining methods was relatively high; however, the heterogeneity between the subgroups was reasonable (*I*^2^ = 41.3%). We believe that acupuncture prescriptions (acupoint combination, method, course of treatment, and stimulus intensity) were the main reasons for heterogeneity.

When comparing acupuncture versus non-acupoint groups, VD rats receiving GV20 + ST36^[30,32,38,39,50]^ (SMD = −1.59, 95% CI: −2.31 to −0.86, *I*^2^ = 59%, *P* < .001) and CV6 + CV12 + CV17 + SP10 + ST36^[26,44]^ (SMD = −2.08, 95% CI: −3.96 to −0.20, *I*^2^ = 80%, *P* = .03) had significantly shortened escape latency. The sensitivity analysis revealed that Zhang et al 2018^[30]^ was the main source of intragroup heterogeneity for the GV20 + ST36 program, which was considered to be caused by the large dispersion of outcome measures in the treatment groups. The intragroup heterogeneity of CV6 + CV12 + CV17 + SP10 + ST36 was mainly due to the inconsistency in the whole course of treatment in the 2 included studies.^[26,44]^ Although there was heterogeneity in both subgroups, deletion of either study within the group did not change the outcome of acupuncture compared to that of the non-acupoint group.

In addition, we performed subgroup analyses according to surgical methods, such as 2VO, 4VO, MCAO, and EO. The results of the RE model indicated that the acupuncture group still had better outcomes than the impaired group; however, compared with subgroups by acupuncture regimens, the subgroup by various surgical methods had increased heterogeneity (Figure S2, Supplemental Digital Content, http://links.lww.com/MD/J111). Therefore, analysis by treatment regimens better reflects the efficacy of interventions.

#### 3.4.2. Number of crossings.

**Figure 5.**
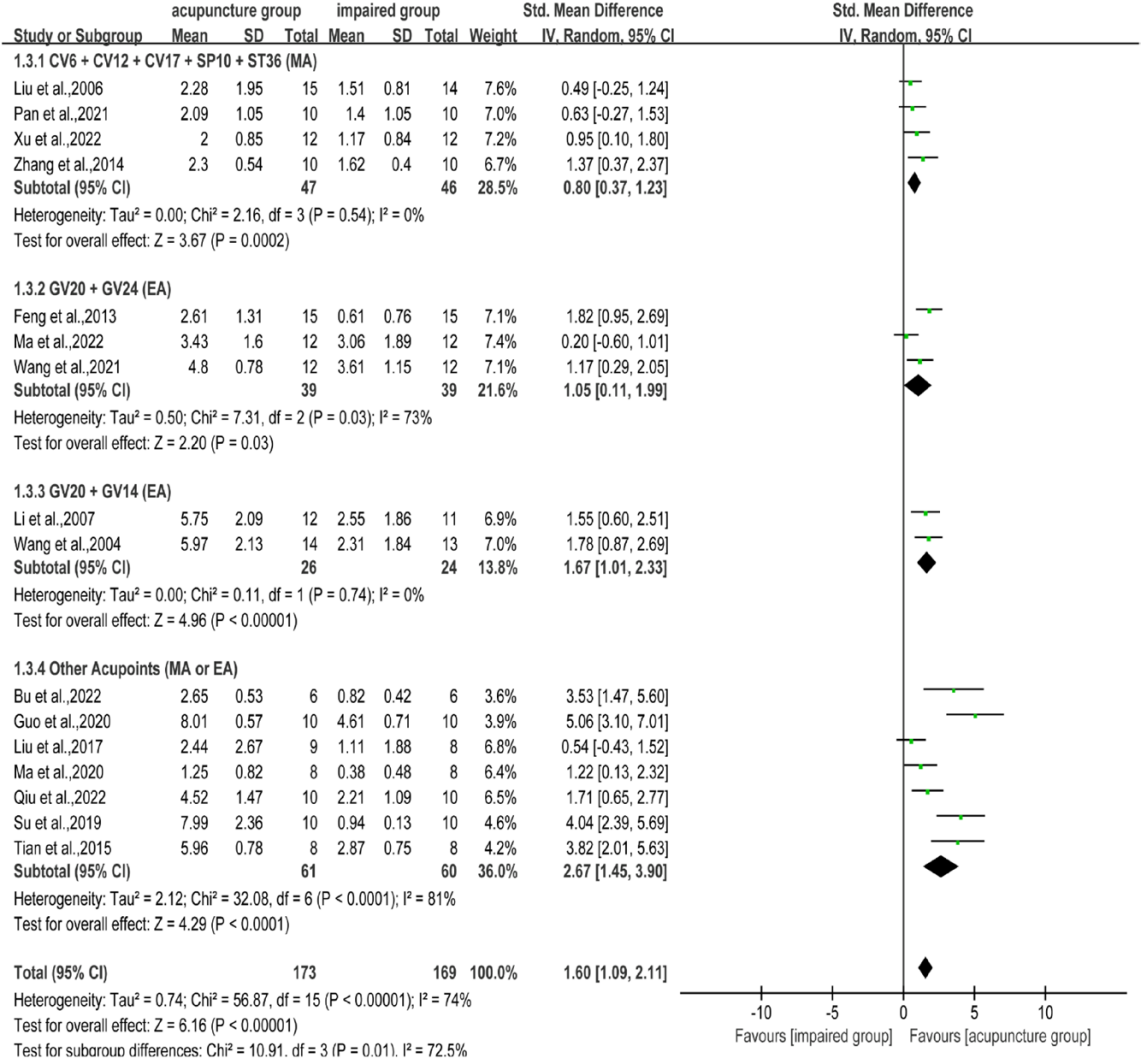
Subgroup analysis of number of crossings between the acupuncture and the impaired groups. Qihai (CV6) + Zhongwan (CV12) + Danzhong (CV17) + bilateral Xuehai (SP10) + bilateral Zusanli (ST36), Baihui (GV20) + Shenting (GV24), Baihui (GV20) + Dazhui (GV14), Other Acupoints (Acupuncture points and methods are different for each study). CI = confidence interval, EA = electroacupuncture, MA = manual acupuncture, SD = standard deviation.

Data from 16 articles showed that acupuncture could significantly increase the number of original platform crossings in VD rats; however, overall heterogeneity could not be ignored (SMD = 1.60, 95% CI: 1.09–2.11, *I*^2^ = 74%, *P* < .001, Fig. 5). In subgroup analyses, the pooled result favored CV17 + CV6 + CV12 + SP10 + ST36^[22,26,44,46]^ (SMD = 0.80, 95% CI: 0.37–1.23, *I*^2^ = 0%, *P* < .001) and GV20 + GV14^[36,47]^ (SMD = 1.67, 95% CI: 1.01–2.33, *I*^2^ = 0%, *P* < .001), whereas GV20 + GV24^[25,33,35]^ (SMD = 1.05, 95% CI: 0.11–1.99, *I*^2^ = 73%, *P* = .03) and other acupoints^[28,31,32,34,48,49,51]^ (SMD = 2.67, 95% CI: 1.45–3.90, *I*^2^ = 81%, *P* < .001) did not show better performance with heterogeneity.

After sensitivity analysis, it was found that the research by Ma et al 2022^[35]^ in the GV20 + GV24 subgroup was the source of heterogeneity. Removing this study from analysis did not influence the stability of the overall results. In the others acupoint group, Bu et al2022,^[34]^ Su et al 2019,^[31]^ Guo et al 2020,^[49]^ and Tian et al 2015^[48]^ were sources of heterogeneity, mainly owing to the reporting of statistics of various acupuncture programs.

#### 3.4.3. Number of Nissl-positive neurons.

**Figure 6.**
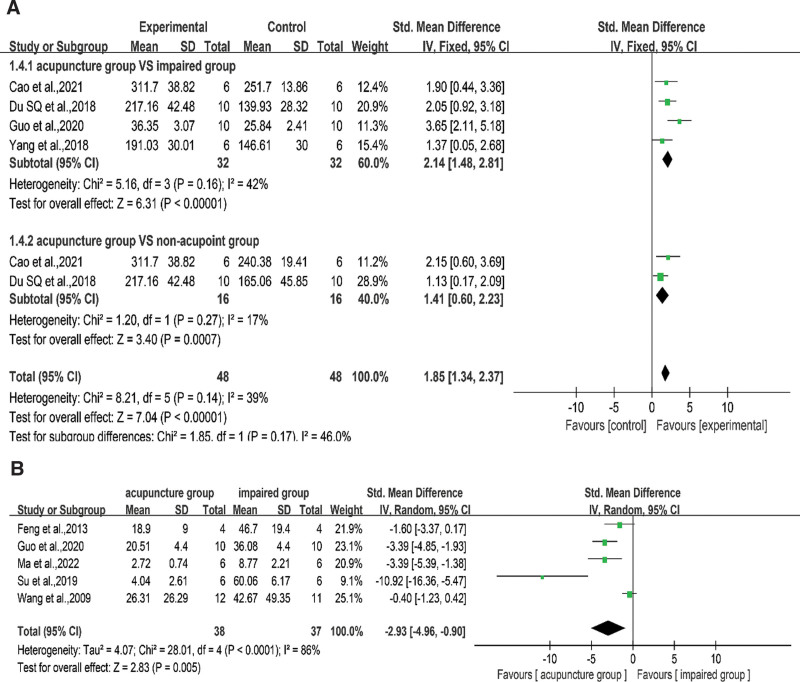
(A) Subgroup analysis of number of Nissl-positive neurons in the acupuncture group compared with the impaired group or non-acupoint group. (B): Analysis of TUNEL-Positive Cell Ratio between the acupuncture and the impaired groups. acupuncture group (experimental); impaired group (control). CI = confidence interval, SD = standard deviation.

Nissl bodies are sensitive indicators of the degree of damage to nerve cells. When neurons are damaged, cytoplasmic Nissl bodies are reduced or eliminated. According to the results summarized by the FE model in Figure 6A, acupuncture can protect Nissl bodies (SMD = 1.85, 95% CI = 1.34–2.37, *I*^2^ = 39%, *P* < .001). Four studies^[29,41,43,49]^ compared Nissl-positive neurons in acupuncture and impaired groups (SMD = 2.14, 95% CI = 1.48–2.81, *I*^2^ = 42%, *P* < .001), and 2 studies^[29,43]^ compared acupuncture and non-acupoints (SMD = 1.41, 95% CI = 0.60–2.23, *I*^2^ = 17%, *P* < .001). Both pooled results showed that acupuncture significantly increased the number of Nissl-positive neurons compared to that of the controls.

#### 3.4.4. TUNEL-positive neurons ratio.

TUNEL staining is an important technique for detecting cell apoptosis, and the degree of cell apoptosis is directly proportional to the number of TUNEL-positive neurons. As shown in Figure 6B, 5 studies^[23,25,31,35,49]^ focused on TUNEL staining. A significant decrease in the TUNEL-positive cell ratio was observed in the acupuncture group compared to that in the impaired group in an RE meta-analysis (SMD = −2.93, 95% CI = −4.96 to −0.90, *I*^2^ = 86%, *P* = .005). The sensitivity analysis was performed to identify heterogeneity sources; the heterogeneity disappeared after the removal of Su et al 2019^[31]^ and Wang et al 2009,^[23]^ and the overall results were unchanged. The source of heterogeneity was the large differences in efficacy between the intervention and control groups, which led to a large degree of dispersion in the measurements.

#### 3.4.5. Oxidative stress (ROS level, MDA level, SOD activity, and GSH-PX activity).

**Figure 7.**
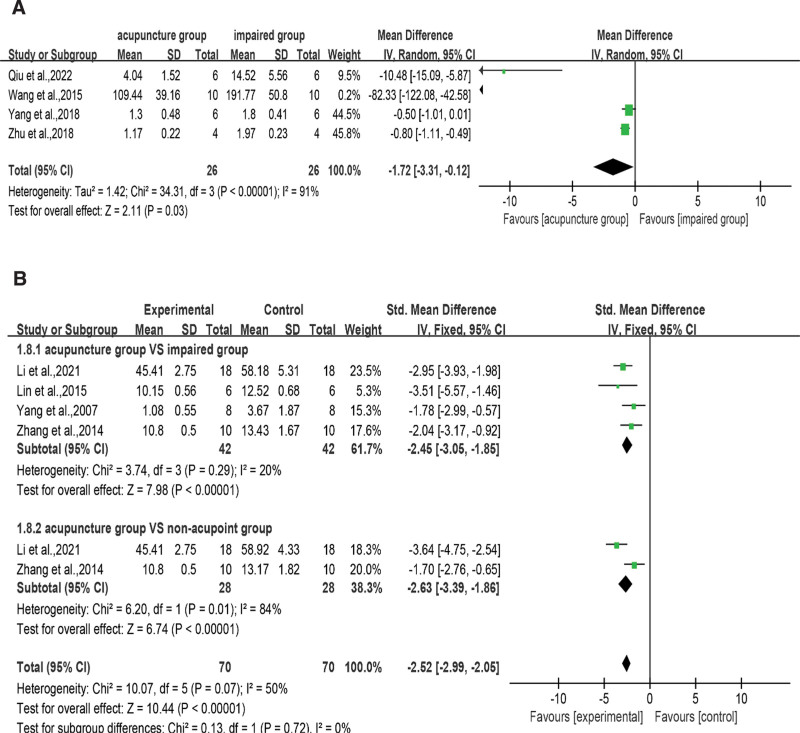
(A) Analysis of ROS expression between the acupuncture and the impaired groups. (B) Subgroup analysis of MDA level in the acupuncture group compared with the impaired group or non-acupoint group. CI = confidence interval, MDA = malondialdehyde amount, ROS = reactive oxygen species, SD = standard deviation.

**Figure 8.**
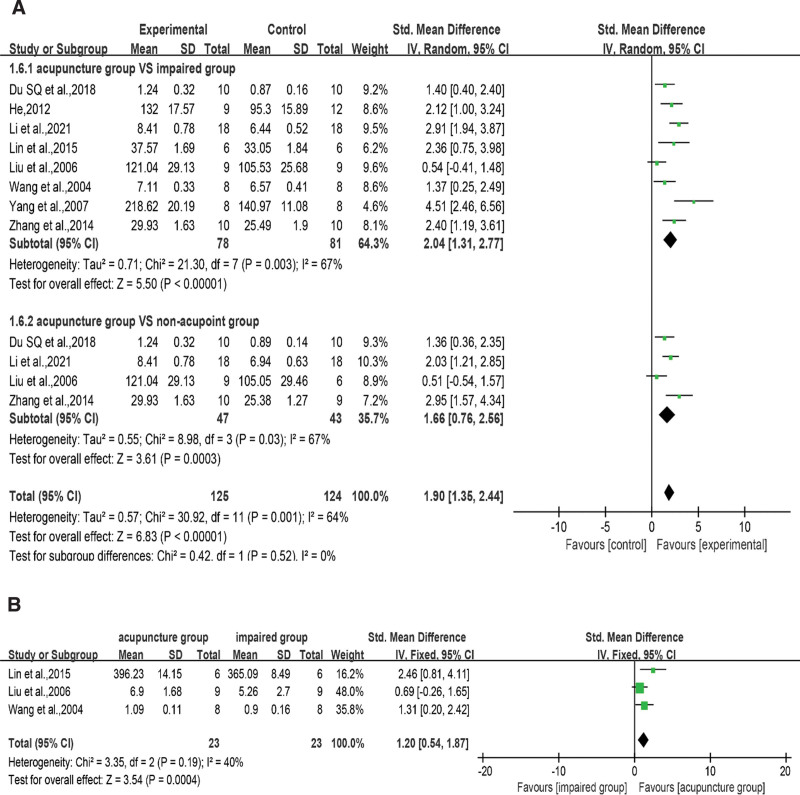
(A) Subgroup analysis of SOD activity in the acupuncture group compared with the impaired group or non-acupoint group. (B) Analysis of GSH-PX activity between the acupuncture and the impaired groups. acupuncture group (experimental); impaired group (control). CI = confidence interval, GSH-PX = glutathione peroxidase, SD = standard deviation, SOD = superoxide dismutase.

SOD, ROS, MDA, and GSH-PX levels are important indicators of oxidative stress. Four literatures^[30,38,41,51]^ recorded the differences in ROS expression between acupuncture and surgical model groups, and Figure 7A showed that acupuncture could inhibit ROS expression (SMD = −1.72, 95% CI = −3.31 to −0.12, *I*^2^ = 91%, *P* < .001). When comparing the MDA content of acupuncture versus impaired^[26,33,50,52]^ and non-acupoint groups,^[26,50]^ significant differences were observed without an apparent degree of heterogeneity, as shown in Figure 7B (SMD = −2.52, 95% CI = −2.99 to −2.05, *I*^2^ = 50%, *P* < .001), indicating that acupuncture significantly reduced MDA levels compared to those of both the impaired (SMD = −2.45, 95% CI = −3.05 to −1.85, *I*^2^ = 20%, *P* < .001) and non-acupoint groups (SMD = −2.63, 95% CI = −3.39 to −1.86, *I*^2^ = 84%, *P* < .001). As shown in Figure 8A, 8 studies^[22,24,26,27,29,47,50,52]^ compared the effects on the SOD level between the acupuncture treatment and control groups (SMD = 1.90, 95% CI = 1.35–2.44, *I*^2^ = 64%, *P* < .001). The subgroup analysis showed that acupuncture showed superior results to those of both the impaired^[22,24,26,27,29,36,50,52]^ (SMD = 2.04, 95% CI = 1.31–2.77, *I*^2^ = 67%, *P* < .001) and non-acupoint groups^[22,26,29,50]^ (SMD = 1.66, 95% CI = 0.76–2.56, *I*^2^ = 67%, *P* < .001); however, both groups had the same heterogeneity (*I*^2^ = 67%). The study by Liu et al 2006^[22]^ was a source of heterogeneity in both groups and overall heterogeneity. After deleting this article, all heterogeneity dropped to approximately 40%. We speculate that the heterogeneity is attributed to the difference in the sample detection technology. As shown in Figure 8B, 3 studies^[22,27,36]^ focused on the GSH-PX level of acupuncture groups compared with that of impaired groups. The pooled results indicated that VD rats that received acupuncture attained more favorable improvements in the GSH-PX level (SMD = 1.20, 95% CI = 0.54–1.87, *I*^2^ = 40%, *P* < .001).

#### 3.4.6. Inflammation (Levels of TNF-α, IL-1β, and IL-6).

**Figure 9.**
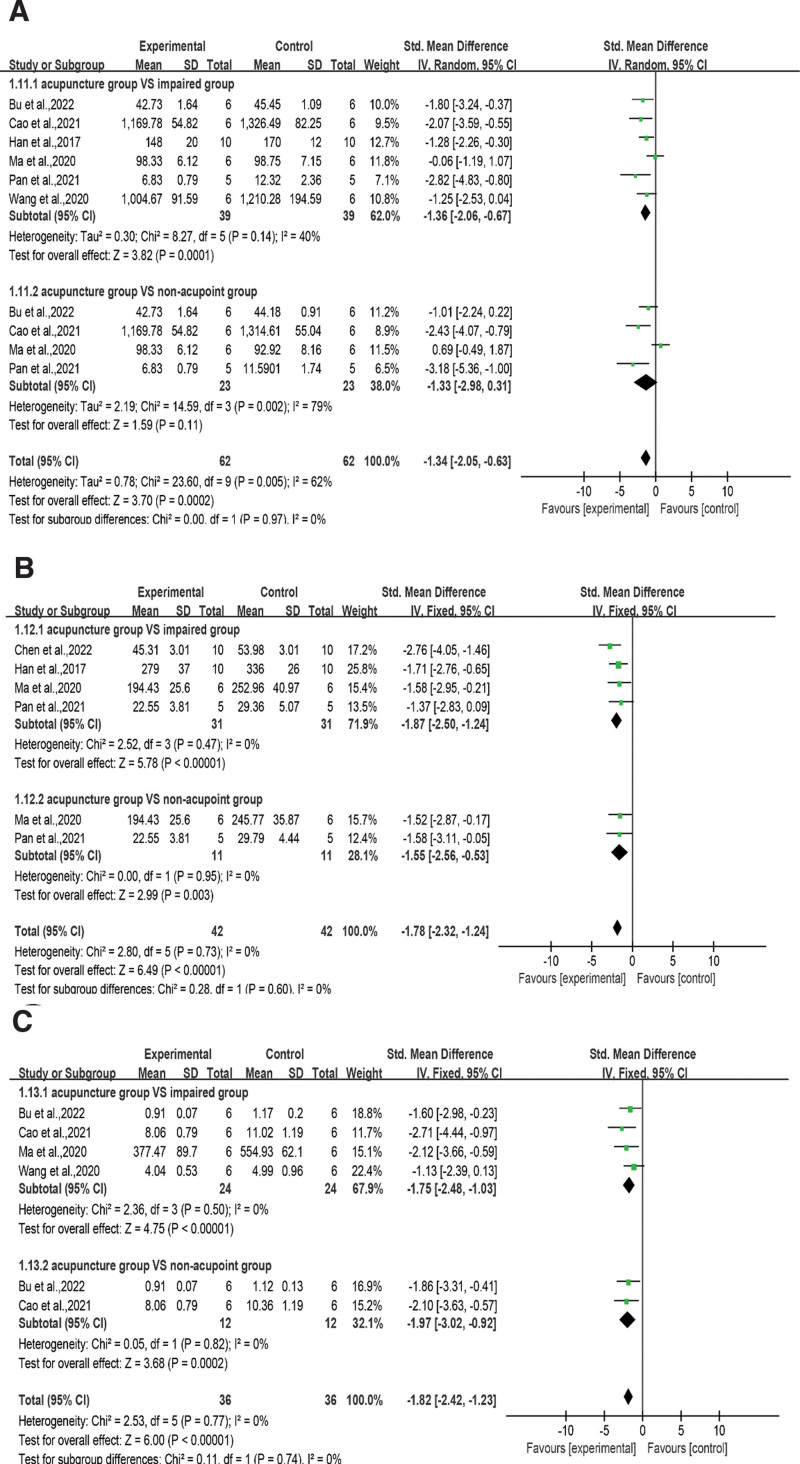
Subgroup analysis of acupuncture versus injury group or non-acupoint group. Abbreviations: A: TNF-α level, B: IL-1β level, C: IL-6 level, (experimental) acupuncture group; (control) impaired group. CI = confidence interval, IL-1β = interleukin-1β, IL-6 = interleukin-6, SD = standard deviation, TNF-α = tumor necrosis factor-α.

Inflammation and oxidative stress are closely related, and inflammation is often involved in the pathological process of VD, downstream of oxidative damage. TNF-α, IL-1β, and IL-6 are the most common neuroinflammatory markers. TNF-α information was available in 6 articles^[32,34,40,42–44]^ (Fig. 9A), and the RE model indicated that rats with acupuncture treatment achieved better TNF-α levels than control rats (SMD = −1.34, 95% CI = −2.05 to −0.63, *I*^2^ = 62%, *P* < .001). In subgroup analysis, acupuncture group had better results than impaired group^[32,34,40,42–44]^ (SMD = −1.36, 95% CI = −2.06 to −0.67, *I*^2^ = 40%, *P* < .001), but there was no difference with the non-acupoint group^[32,34,43,44]^ (SMD = −1.33, 95% CI = −2.98 to 0.31, *I*^2^ = 79%, *P* = .11). The overall pooled heterogeneity was derived from the acupuncture versus non-acupoint groups. In non-acupoint group, Ma et al 2020^[32]^ used white matter as the test object, which differed from the hippocampus test used in other studies. Their team believed that acupuncture cannot significantly change the TNF-α content in the white matter of rats with VD. As shown in Figure 9B, the overall results of IL-1β in the acupuncture versus surgery group and the non-acupoint group were pooled from 4 studies^[32,40,44,45]^ (SMD = −1.78, 95% CI = −2.32 to −1.24, *I*^2^ = 0%, *P* < .001). The results within subgroups showed that acupuncture had better results than both the impaired group^[32,40,44,45]^ (SMD = −1.87, 95% CI = −2.50 to −1.24, *I*^2^ = 0%, *P* < .001) and the non-acupoint group^[32,44]^ (SMD = −1.55, 95% CI = −2.56 to −0.53, *I*^2^ = 0%, *P* = .003). As shown in Figure 9C, the results of acupuncture on IL-6 pooled in 4 studies^[32,34,42,43]^ under the FE model showed the same trend (SMD = −1.82, 95% CI = −2.42 to −1.23, *I*^2^ = 0%, *P* < .001). There was no heterogeneity within or between subgroups, and the analysis showed that the acupuncture group not only had better results than the impaired group^[32,34,42,43]^ (SMD = −1.75, 95% CI = −2.48 to −1.03, *I*^2^ = 0%, *P* < .001) but also the non-acupoint group^[34,43]^ (SMD = −1.97, 95% CI = −3.02 to −0.92, *I*^2^ = 0%, *P* < .001).

### 3.5. Publication bias

**Figure 10.**
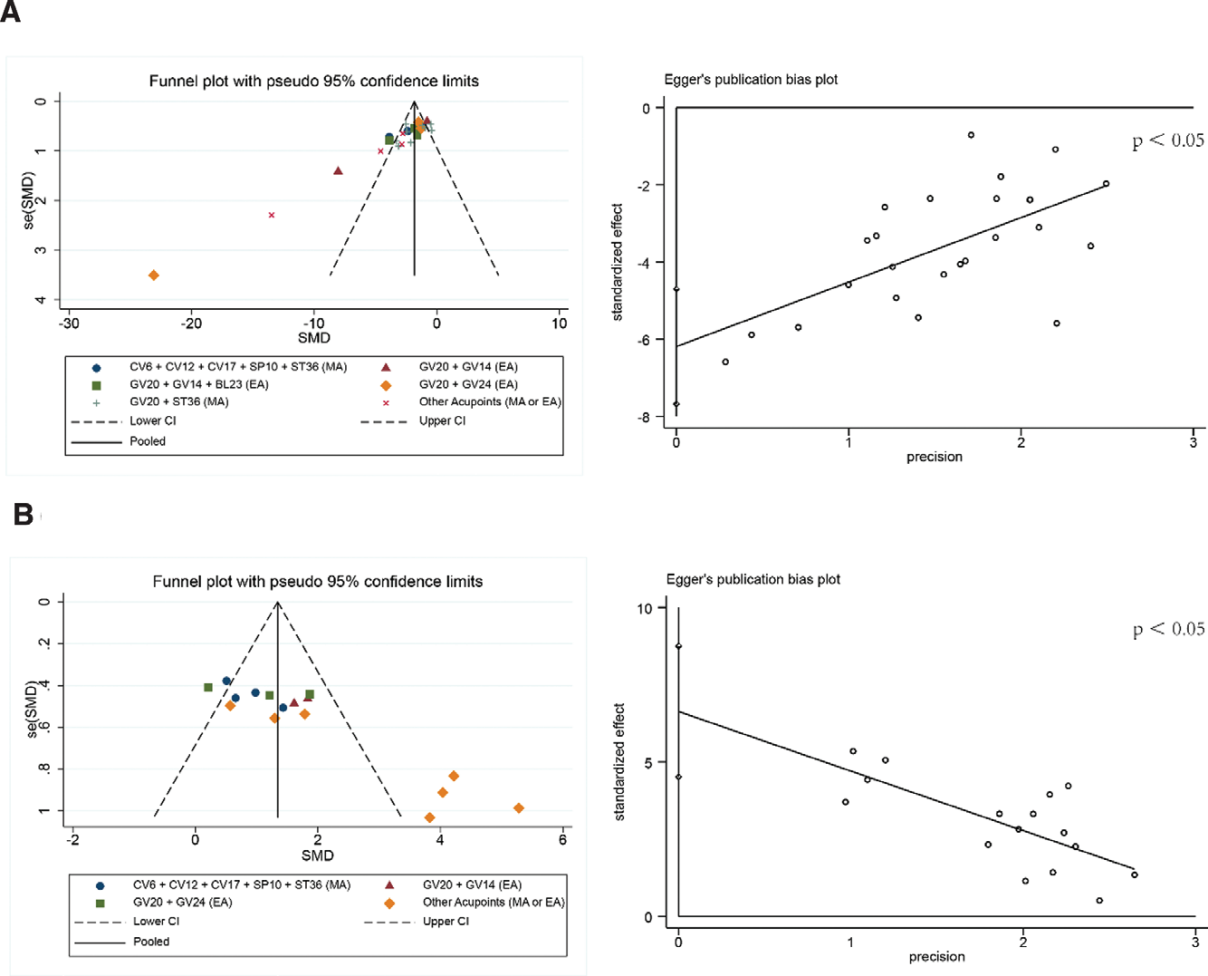
Publication bias in escape latency and number of crossings. A: escape latency, B: number of crossings. EA = electroacupuncture, MA = manual acupuncture, SMD = standard mean difference.

On the basis of escape latency and number of crossings, acupuncture versus impaired groups were compared using funnel plots and Egger’s tests. Figure 10A shows that some studies were outside the inverted funnel, and Egger’s test *P* was less than .05, indicating publication bias in the escape latency outcome. After subgroup analysis, it was found that the bias mainly came from 6 trials,^[31,33,40,46,48,51]^ mainly because the acupuncture programs (acupoint combination, acupuncture method, stimulation intensity) differed. A publication bias was also evident in the number of original platform crossings (Fig. 10B), mainly in the other acupoint group.^[31,34,48,49]^ The reason for this bias is that the acupuncture protocols of all the studies in this group were different. Additionally, Ma et al 2022^[35]^ was sources of bias since this research concluded that acupuncture had no significant impact on total number of original platform crossings in comparison to impaired group. Although both outcomes were biased, we found the bias and removed it uniformly, and the results were still stable (Figures S3 and S4, Supplemental Digital Content, http://links.lww.com/MD/J112).

## 4. Discussion

### 4.1. Summary of evidence

Acupuncture has shown significant therapeutic effects in animal models of VD, but scattered evidence and controversy over its efficacy have affected the strength of the evidence for acupuncture. Our research aimed to show that acupuncture can significantly improve the cognitive ability of animal models of VD, and acupuncture was better than the impaired and non-acupoint groups in alleviating the mechanisms of oxidative stress, inflammation, and apoptosis. In view of this, A systematic review and the first meta-analysis of the effectiveness of acupuncture at a specific mechanistic level were conducted. Thirty-one eligible studies involving 751 rats were included in the study. In the results, it was found that compared with those of the impaired and non-acupoint groups, acupuncture significantly improved the Morris water maze results in VD rats; compared with those of the impaired and non-acupoint groups, acupuncture significantly increased the number of Nissl-positive neurons, and the proportion of TUNEL-positive neurons was also lower in the acupuncture group than in the impaired group; acupuncture significantly increased the activity of SOD and GSH-PX and reduced the MDA content; In contrast to the impaired group, the acupuncture group has a significantly lower content of TNF-α levels, and although the acupuncture group and non-acupoints did not have any significant differences, there was a tendency to reduce the TNF-α content; and Compared with those of the impaired and non-acupoint groups, the acupuncture group had significantly reduced levels of IL-1β and IL-6. There was mild heterogeneity among the 31 studies that were considered upper-middle quality. Subgroup analysis and sensitivity analysis were used to identify heterogeneity sources; although a small number of studies were heterogeneous, the overall results remained stable.

### 4.2. Possible mechanisms

The pathogenesis of VD is largely influenced by oxidative stress, inflammation, and apoptosis.^[53]^ These 3 factors are interrelated and inseparable. Oxidative stress often occurs upstream and has an important impact on pathological processes like inflammation and apoptosis.^[14]^ As part of this study, we summarize the potential mechanisms by which acupuncture can prevent VD. First, acupuncture suppresses oxidative stress.^[22,24,26,27,29–31,36–39,41,50–52]^ Oxidative stress is the most direct response mechanism in cerebral ischemia and hypoxia. A large number of lipid peroxidation products and free radicals will be produced when cerebral blood flow slows and oxygen content is insufficient, resulting in increased cell membrane permeability, causing excessive edema and excitatory transmitter release, irreversible neuronal damage, and new infarcts after accumulation of necrotic neurons.^[54]^ Acupuncture can increase cerebral blood flow and the number of Nissl nerve sources; reverse mitochondrial damage; reduce infarct size; upregulate SOD, GSH-PX, ATP, COX, amyloid precursor protein, and acetylcholine levels; and downregulate reactive oxygen species, MDA, nitric oxide, and inducible nitric oxide synthase levels. Signaling pathways PI3K/AKT/mTOR and Nrf2/ARE/HO-1 are mainly responsible for these functions. Second, acupuncture inhibits neuronal inflammation.^[28,29,31,32,34,36,38,40,42–46,51]^ Inflammation is a downstream effect of oxidative stress that contributes to the development of nervous system diseases. When cerebral ischemia and hypoxia occur, inflammation is induced, and a large number of pro-inflammatory cytokines accumulate, causing an inflammatory storm and damage to neurons.^[55]^ Acupuncture can protect damaged neurons by upregulating IL-4 and IL-10 levels and downregulating TNF-α, IL-1β, IL-6, IL-2, Iba-1, and NLRP3 levels. These anti-inflammatory mechanisms of acupuncture are mainly attributed to the JAK2/STAT3 or TLR4/MyD88/NF-kB signaling pathway. Third, acupuncture reduces apoptosis.^[23,25,29–31,33,35,48,49,51]^ Oxidative stress induces apoptosis and can also accelerate the death of hypoxic neurons. After brain hypoxia, the mitochondria release apoptotic proteins into the cytoplasm. Apoptosis is initiated when pro-apoptotic proteins accumulate to a certain extent.^[56]^ Cell survival is maintained by a dynamic balance between apoptotic factors and apoptosis. When chronic continuous hypoxia occurs, caspases, Bax, and other pro-apoptotic factors are activated and released in large quantities, and the content of anti-apoptotic inhibitors such as Bcl-1 and Bcl-2 decreases, disrupting the original cellular homeostasis.^[57]^ Acupuncture can reduce the proportion of TUNEL-positive cells by upregulating Bcl-1 and Bcl-2 expression and downregulating the levels of BAX, FAX, P53, caspase 3, and caspase 8, which is mainly mediated via the PI3K/AKT/mTOR or Trx-1/P-ASK/P-P38/P-JNK signaling pathway.

### 4.3. Implications

The ultimate purpose of animal research is to guide clinical practice and improve the accuracy and efficiency of clinical treatments.^[58]^ Scientific research on acupuncture has unique characteristics. It is used to test acupuncture methods, which have been widely used for some clinical diseases and then in animals. Understanding the therapeutic mechanism of acupuncture can promote the development of acupuncture in clinical practice. The role of neuroimaging in the diagnosis of VD cannot be overstated with the help of imaging techniques to visualize lesions in patients’ brains, such as subcortical infarction, hippocampal atrophy, and other abnormalities. Clinical and animal studies have confirmed that acupuncture can reorganize brain regions related to language and cognition, such as the frontal, temporal, parietal, and occipital lobes, where acupuncture points and stimulation volume are important factors in brain neuroplasticity.^[59]^ It has been reported that the levels of peripheral oxidative stress biomarkers in patients with VD increase and the antioxidant capacity decreases.^[60]^ Although there is no direct report that acupuncture can improve oxidative stress in patients with VD, we believe that this is also an important mechanism of acupuncture in clinical practice. Fortunately, studies have shown that treatment with “San Jiao” acupuncture (CV6 + CV12 + CV17 + SP10 + ST36 + SJ5) can significantly increase the levels of anti-inflammatory substances, such as T lymphocytes, in the peripheral blood and significantly decrease the amount of pro-inflammatory factors, such as TNF-α, in the blood, in addition to a decreasing trend in the levels of IL-1β and IL-2.^[61]^ The conclusions drawn from acupuncture to improve VD animal models are just effective in promoting clinical practice. In the future, more clinical and animal studies will be required to confirm the efficacy and advantages of acupuncture. Our study is closely linked to the clinical reality of neuronal death, oxidative stress, and cellular inflammation, and the conclusions drawn may provide a reference for countries that are hesitant to consider introducing acupuncture into the clinical treatment of VD.

### 4.4. Strengths

**Figure 11.**
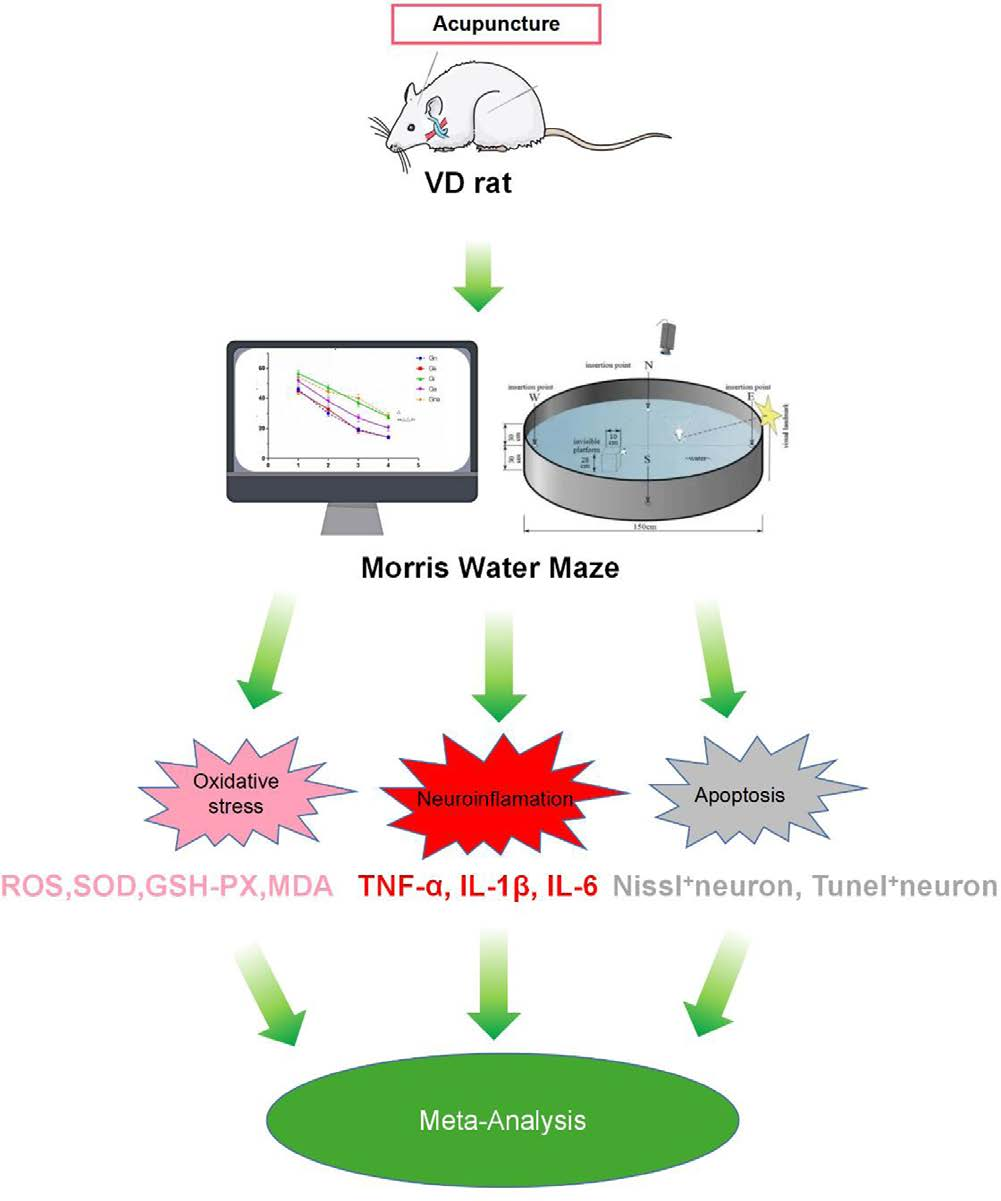
The advantages and innovation of this research. GSH-PX = glutathione peroxidase, IL-1β = interleukin-1β, IL-6 = interleukin-6, MDA = malondialdehyde amount, ROS = reactive oxygen species, SOD = superoxide dismutase, TNF-α = tumor necrosis factor-α, VD = vascular dementia.

To our knowledge, there have been 3 systematic reviews of acupuncture improving cognition in animals.^[62–64]^ Our findings are similar to those of previous studies on the Morris water maze, yet compensate for their deficiencies. As previous studies have only revealed behavioral performance,^[63,64]^ we analyzed the number and proportion of neurons from stained sections and compared specific markers for oxidative stress and neuronal inflammation (Fig. 11). However, noteworthily, the 2 previous systematic reviews are now out of date, Studies included in the analysis were relatively few, and the sham acupuncture, drug, and impaired groups were collectively referred to as the control group.^[63]^ In addition, the outcome of one study was mixed by VD, Alzheimer’s disease, Parkinson’s disease, and other diseases that can lead to cognitive abnormalities.^[62]^ The present study updated the included literature to separate sham acupuncture from impaired acupuncture, and the evidence for the results obtained was stronger.

### 4.5. Limitations

Limitations of this study exist. First, All the experiments included in this study were carried out in China, which led to the existence of implementation bias. Second, The included articles did not carry out allocation concealment when carrying out animal experiments, and a few of them carried out blind implementation. These shortcomings will cause selection bias and result measurement bias. Third, Animal experiments have a single modeling method and simple pathogenesis, while clinical VD patients have various pathogenic causes and complex pathogenesis. Therefore, the gap between animal experiments and clinical application needs to be paid attention to.

### 4.6. Looking forward to the future

High-quality research methods in animal research can facilitate their translation to clinical research. At present, the scientific research of many acupuncture research teams is still in its infancy, and research methods could be improved greatly.^[10]^ Based on our findings, many researchers’ experimental designs and reporting of experimental results have not been standardized well enough. We recommend that animal studies follow the ARRIVE guidelines (Animal Research: Reporting of In Vivo Experiments).^[65]^ In addition, there have been improvements in the selection of animal models. Although the current mainstream surgical methods can model VD animals, the human pathological mechanism is complex.^[66]^ At this point, it is necessary to consider the combination of different models to create an animal model of VD that is closer to the condition seen in humans.^[67]^ From another perspective, similar to the targeting of drugs, acupuncture treatment in the clinic should identify syndrome types of traditional Chinese medicine (TCM). The conclusions drawn from acupuncture treatment performed using a common modeling approach may affect the actual efficacy of acupuncture when applied directly in the clinic. Therefore, we suggest that the disease-syndrome combination model can be used to simulate TCM syndrome types, which may better reflect the targeting of acupuncture treatment.^[68]^ Finally, our hope is that more high-quality preclinical studies will confirm this study’s conclusions and more systematic medical insurance coverage to promote the clinical application of acupuncture in various countries, bringing a new dawn to the recovery of patients around the world.

## 5. Conclusion

This meta-analysis found that acupuncture can shorten the escape latency and increase the number of crossings of VD rats. The internal mechanism of acupuncture effect is to inhibit oxidative stress, neuroinflammation, and cell apoptosis in the brain of VD rats. The conclusion of this study can show that the efficacy of acupuncture is clear and that acupuncture is not a placebo effect. Nevertheless, attention needs to be paid to the gap between animal experiments and clinical applications.

## Acknowledgments

Thanks to Gaofeng Liu and Chengwei Fu for their efforts in this research.

## Author contributions

**Conceptualization:** Qin Wen.

**Formal analysis:** Xueqin Hong, Kunze He.

**Funding acquisition:** Qin Wen.

**Investigation:** Xueqin Hong, Kunze He.

**Methodology:** Xueqin Hong, Kunze He.

**Supervision:** Min Li.

**Writing – original draft:** Qin Wen.

**Writing – review & editing:** Buping Liu, Min Li.

## Supplementary Material






