# Supplementary material for: Can acupuncture reverse oxidative stress and neuroinflammatory damage in animal models of vascular dementia?: A preclinical systematic review and meta-analysis
Source: Medicine (Baltimore). 2023 Jun 9;102(23):e33989. doi: 10.1097/MD.0000000000033989 (PMC10256398; doi:10.1097/MD.0000000000033989)
Supplement: Supplementary file 1 [file medi-102-e33989-s001.pdf]

Figure 1. Risk of bias summary

	Random sequence generation (selection bias)	Baseline characteristics (selection bias)	Allocation concealment (selection bias)	Random housing (performance bias)	Blinding of participants and personnel (performance bias)	Blinding of outcome assessment (detection bias)	Binding (detection bias)	Incomplete outcome data (attrition bias)	Selective reporting (reporting bias)	Other bias
Bu et al.,2022	+	+	?	+	?	+	?	+	+	+
Cao et al.,2021	+	+	?	+	?	+	+	+	+	?
Chen et al.,2022	+	+	?	+	?	+	?	+	+	+
Du SQ et al.,2018	+	+	?	+	?	+	?	+	+	+
Feng et al.,2013	+	+	?	+	?	+	?	+	+	+
Guo et al.,2020	+	+	?	+	?	+	?	+	+	+
Han et al.,2017	+	+	?	+	?	?	?	+	+	+
He,2012	+	+	?	+	?	+	?	+	+	?
Li et al.,2007	+	+	?	?	?	+	?	+	+	?
Li et al.,2016	+	+	?	+	?	?	?	+	+	+
Li et al.,2021	+	+	?	+	?	+	?	+	+	?
Lin et al.,2015	+	+	?	+	?	?	?	+	+	+
Liu et al.,2006	+	+	?	+	?	?	?	+	+	+
Liu et al.,2017	+	+	?	+	?	?	?	+	+	+
Ma et al.,2020	+	+	?	+	?	+	?	+	+	+
Ma et al.,2022	+	+	?	+	?	+	?	+	+	+
Pan et al.,2021	+	+	?	+	?	+	?	+	+	?
Qiu et al.,2022	+	+	?	+	?	+	?	+	+	+
Su et al.,2019	+	+	?	?	?	+	?	+	+	?
Tian et al.,2015	+	+	?	+	?	+	?	+	+	+
Wang et al.,2004	+	+	?	?	?	+	?	+	+	+
Wang et al.,2009	+	+	?	+	?	+	?	+	+	+
Wang et al.,2015	+	+	?	+	?	+	?	+	+	?
Wang et al.,2020	+	+	?	+	?	?	+	+	+	?
Wang et al.,2021	+	+	?	+	?	+	?	+	+	?
Xu et al.,2022	+	+	?	+	?	+	?	+	+	+
Yang et al.,2007	+	+	?	?	?	+	?	+	+	+
Yang et al.,2018	+	+	?	+	?	+	+	+	+	+
Zhang et al.,2014	+	+	?	+	?	?	?	+	+	?
Zhu et al.,2013	+	+	?	+	?	+	−	+	+	+
Zhu et al.,2018	+	+	?	+	?	+	?	+	+	?
