# Supplementary material for: Can acupuncture reverse oxidative stress and neuroinflammatory damage in animal models of vascular dementia?: A preclinical systematic review and meta-analysis
Source: Medicine (Baltimore). 2023 Jun 9;102(23):e33989. doi: 10.1097/MD.0000000000033989 (PMC10256398; doi:10.1097/MD.0000000000033989)
Supplement: Supplementary file 2 [file medi-102-e33989-s002.pdf]

Figure 2. Subgroup analysis of escape latency in acupuncture versus impaired groups based on different models

Study or Subgroup	Experimental Mean	Experimental SD	Experimental Total	Control Mean	Control SD	Control Total	Weight	Std. Mean Difference IV, Random, 95% CI
**1.2.1 2VO**								
Cao et al.,2021	28.6	13.4	5	55.94	12.23	5	3.8%	-1.92 [-3.57, -0.28]
Chen et al.,2022	32.29	4.81	10	42.82	6.64	10	4.6%	-1.74 [-2.80, -0.68]
Du SQ et al.,2018	18.81	11.69	10	35.87	17.08	10	4.7%	-1.12 [-2.07, -0.16]
Guo et al.,2020	11.83	2.59	10	24.09	5.66	10	4.3%	-2.67 [-3.94, -1.40]
Han et al.,2017	10.7	2.09	10	30.12	2.7	10	2.4%	-7.70 [-10.51, -4.90]
Li et al.,2016	21.11	8.64	8	50.83	9.43	8	3.9%	-3.11 [-4.69, -1.53]
Li et al.,2021	26.01	6.61	18	46.06	9.04	18	4.8%	-2.48 [-3.37, -1.58]
Ma et al.,2020	17.56	6.42	8	48.46	45.59	8	4.6%	-0.90 [-1.94, 0.15]
Pan et al.,2021	20.35	5.76	10	27.33	6.24	10	4.7%	-1.11 [-2.07, -0.16]
Qiu et al.,2022	32.42	3.7886	10	47.58	4.0529	10	3.9%	-3.70 [-5.25, -2.15]
Wang et al.,2015	21.48	8.82	10	46.43	11.29	10	4.4%	-2.36 [-3.56, -1.16]
Wang et al.,2020	27.94	21.99	6	36.19	17.6	6	4.5%	-0.38 [-1.53, 0.76]
Xu et al.,2022	20.83	3.17	12	34.4	3.81	12	4.1%	-3.74 [-5.15, -2.33]
Yang et al.,2018	11.13	10.17	6	38.83	7.46	6	3.6%	-2.87 [-4.67, -1.07]
Zhu et al.,2013	10.28	4.27	6	25.78	13.02	6	4.2%	-1.48 [-2.82, -0.14]
Zhu et al.,2018	42.29	54.16	10	65.28	37.84	10	4.8%	-0.47 [-1.36, 0.42]
**Subtotal (95% CI)**			**149**			**149**	**67.4%**	**-2.10 [-2.72, -1.49]**
Heterogeneity: Tau² = 1.14; Chi² = 60.36, df = 15 (P < 0.00001); I² = 75%								
Test for overall effect: Z = 6.67 (P < 0.00001)								
**1.2.2 4VO**								
Bu et al.,2022	17.25	5.75	6	69.97	25.4	6	3.7%	-2.64 [-4.36, -0.93]
Li et al.,2007	10.85	5.94	12	46.51	34.49	11	4.7%	-1.42 [-2.36, -0.49]
Tian et al.,2015	45.59	5.33	8	73.42	6.71	8	3.3%	-4.34 [-6.33, -2.35]
Wang et al.,2004	29.89	32.62	14	58.08	38.77	13	4.9%	-0.77 [-1.55, 0.02]
**Subtotal (95% CI)**			**40**			**38**	**16.7%**	**-2.03 [-3.30, -0.76]**
Heterogeneity: Tau² = 1.21; Chi² = 12.88, df = 3 (P = 0.005); I² = 77%								
Test for overall effect: Z = 3.13 (P = 0.002)								
**1.2.3 MCAO**								
Feng et al.,2013	20.5	30.98	15	57.5	16.46	15	4.9%	-1.45 [-2.27, -0.63]
Liu et al.,2017	26.74	21.54	9	52.84	19.38	8	4.6%	-1.20 [-2.26, -0.15]
Su et al.,2019	4.06	2.25	10	59.94	5.41	10	1.3%	-12.92 [-17.47, -8.37]
Wang et al.,2021	40.92	0.88	12	59.18	0.69	12	0.6%	-22.30 [-29.24, -15.35]
**Subtotal (95% CI)**			**46**			**45**	**11.4%**	**-7.11 [-10.91, -3.31]**
Heterogeneity: Tau² = 11.77; Chi² = 58.26, df = 3 (P < 0.00001); I² = 95%								
Test for overall effect: Z = 3.67 (P = 0.0002)								
**1.2.4 EO**								
Zhang et al.,2014	18.03	4.6	10	30.54	5.89	10	4.4%	-2.27 [-3.44, -1.09]
**Subtotal (95% CI)**			**10**			**10**	**4.4%**	**-2.27 [-3.44, -1.09]**
Heterogeneity: Not applicable								
Test for overall effect: Z = 3.78 (P = 0.0002)								
**Total (95% CI)**			**245**			**242**	**100.0%**	**-2.34 [-2.93, -1.74]**
Heterogeneity: Tau² = 1.69; Chi² = 133.48, df = 24 (P < 0.00001); I² = 82%								
Test for overall effect: Z = 7.73 (P < 0.00001)								
Test for subgroup differences: Chi² = 6.58, df = 3 (P = 0.09), I² = 54.4%								

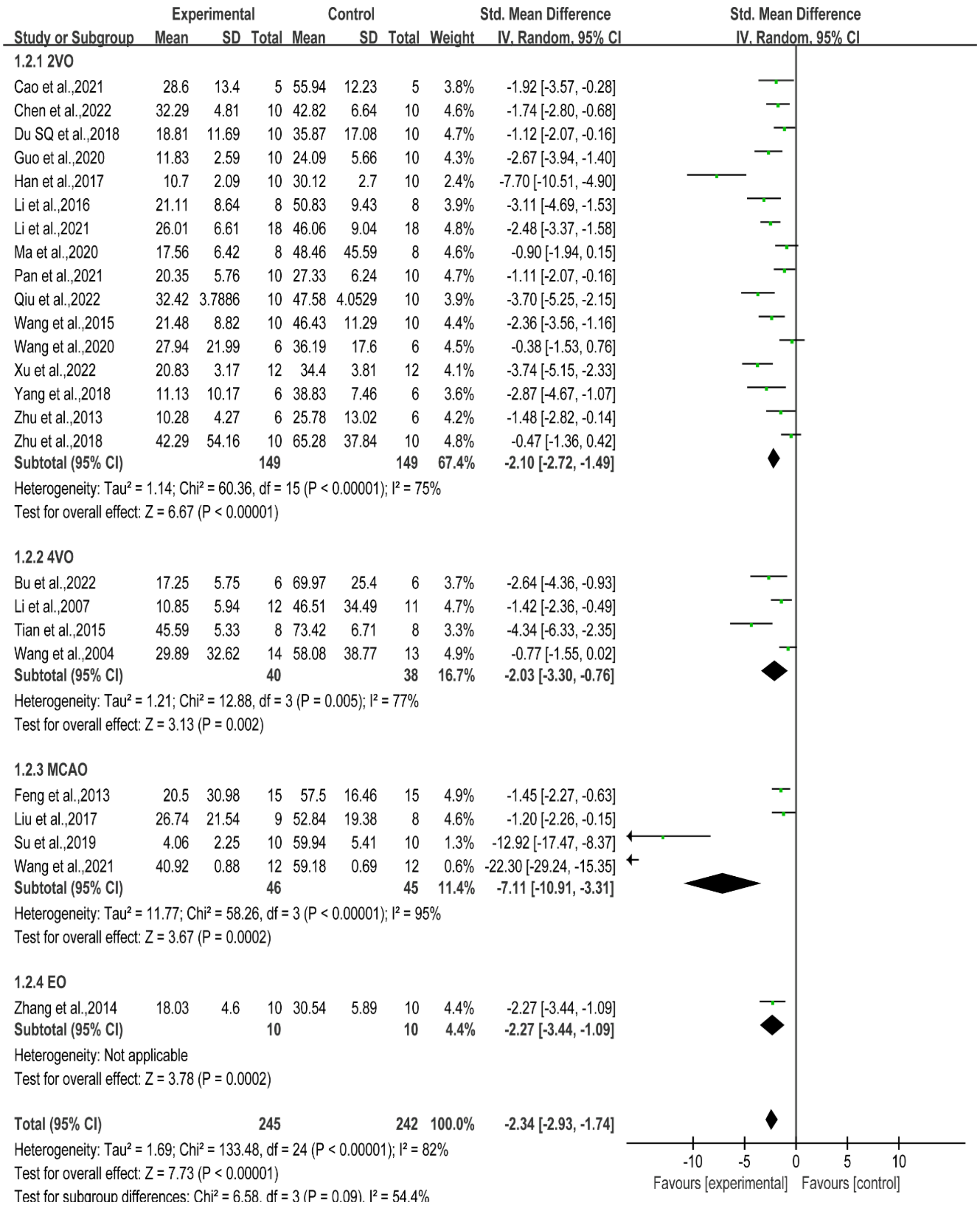

Abbreviations: SD rat (Sprague–Dawley rat); 2VO (bilateral common carotid artery occlusion); 4VO (4-vessel occlusion); MCAO (middle cerebral artery occlusion).
