# Supplementary material for: Can acupuncture reverse oxidative stress and neuroinflammatory damage in animal models of vascular dementia?: A preclinical systematic review and meta-analysis
Source: Medicine (Baltimore). 2023 Jun 9;102(23):e33989. doi: 10.1097/MD.0000000000033989 (PMC10256398; doi:10.1097/MD.0000000000033989)
Supplement: Supplementary file 3 [file medi-102-e33989-s003.pdf]

Figure 3. Sensitivity analysis of escape latency

	acupuncture group			impaired group				Std. Mean Difference
Study or Subgroup	Mean	SD	Total	Mean	SD	Total	Weight	IV, Random, 95% CI
**1.1.1 GV20 + ST36 (MA)**								
Cao et al.,2021	28.6	13.4	5	55.94	12.23	5	3.3%	-1.92 [-3.57, -0.28]
Du SQ et al.,2018	18.81	11.69	10	35.87	17.08	10	6.2%	-1.12 [-2.07, -0.16]
Li et al.,2016	21.11	8.64	8	50.83	9.43	8	3.5%	-3.11 [-4.69, -1.53]
Li et al.,2021	26.01	6.61	18	46.06	9.04	18	6.6%	-2.48 [-3.37, -1.58]
Ma et al.,2020	17.56	6.42	8	48.46	45.59	8	5.7%	-0.90 [-1.94, 0.15]
Wang et al.,2015	21.48	8.82	10	46.43	11.29	10	4.9%	-2.36 [-3.56, -1.16]
Wang et al.,2020	27.94	21.99	6	36.19	17.6	6	5.2%	-0.38 [-1.53, 0.76]
Yang et al.,2018	11.13	10.17	6	38.83	7.46	6	2.9%	-2.87 [-4.67, -1.07]
Zhu et al.,2018	42.29	54.16	10	65.28	37.84	10	6.6%	-0.47 [-1.36, 0.42]
**Subtotal (95% CI)**			**81**			**81**	**44.8%**	**-1.62 [-2.29, -0.95]**
Heterogeneity: Tau² = 0.67; Chi² = 23.68, df = 8 (P = 0.003); I² = 66%								
Test for overall effect: Z = 4.74 (P < 0.00001)								
**1.1.2 GV20 + GV24 (EA)**								
Feng et al.,2013	20.5	30.98	15	57.5	16.46	15	7.1%	-1.45 [-2.27, -0.63]
Liu et al.,2017	26.74	21.54	9	52.84	19.38	8	5.6%	-1.20 [-2.26, -0.15]
Wang et al.,2021	40.92	0.88	12	59.18	0.69	12	0.0%	-22.30 [-29.24, -15.35]
**Subtotal (95% CI)**			**24**			**23**	**12.7%**	**-1.36 [-2.01, -0.71]**
Heterogeneity: Tau² = 0.00; Chi² = 0.13, df = 1 (P = 0.72); I² = 0%								
Test for overall effect: Z = 4.12 (P < 0.0001)								
**1.1.3 GV20 + GV14 (EA)**								
Han et al.,2017	10.7	2.09	10	30.12	2.7	10	0.0%	-7.70 [-10.51, -4.90]
Li et al.,2007	10.85	5.94	12	46.51	34.49	11	6.3%	-1.42 [-2.36, -0.49]
Wang et al.,2004	29.89	32.62	14	58.08	38.77	13	7.3%	-0.77 [-1.55, 0.02]
**Subtotal (95% CI)**			**26**			**24**	**13.6%**	**-1.04 [-1.68, -0.41]**
Heterogeneity: Tau² = 0.02; Chi² = 1.11, df = 1 (P = 0.29); I² = 10%								
Test for overall effect: Z = 3.22 (P = 0.001)								
**1.1.4 GV20 + GV14 + BL23 (EA)**								
Chen et al.,2022	32.29	4.81	10	42.82	6.64	10	5.6%	-1.74 [-2.80, -0.68]
Qiu et al.,2022	32.42	3.79	10	47.58	4.05	10	0.0%	-3.70 [-5.25, -2.15]
Zhu et al.,2013	10.28	4.27	6	25.78	13.02	6	4.3%	-1.48 [-2.82, -0.14]
**Subtotal (95% CI)**			**16**			**16**	**9.9%**	**-1.64 [-2.47, -0.80]**
Heterogeneity: Tau² = 0.00; Chi² = 0.09, df = 1 (P = 0.76); I² = 0%								
Test for overall effect: Z = 3.85 (P = 0.0001)								
**1.1.5 CV6 + CV12 + CV17 + SP10 + ST36 (MA)**								
Pan et al.,2021	20.35	5.76	10	27.33	6.24	10	6.2%	-1.11 [-2.07, -0.16]
Xu et al.,2022	20.83	3.17	12	34.4	3.81	12	0.0%	-3.74 [-5.15, -2.33]
Zhang et al.,2014	18.03	4.6	10	30.54	5.89	10	5.0%	-2.27 [-3.44, -1.09]
**Subtotal (95% CI)**			**20**			**20**	**11.2%**	**-1.64 [-2.76, -0.51]**
Heterogeneity: Tau² = 0.37; Chi² = 2.22, df = 1 (P = 0.14); I² = 55%								
Test for overall effect: Z = 2.85 (P = 0.004)								
**1.1.6 Other Acupoints (MA or EA)**								
Bu et al.,2022	17.25	5.75	6	69.97	25.4	6	3.1%	-2.64 [-4.36, -0.93]
Guo et al.,2020	11.83	2.59	10	24.09	5.66	10	4.6%	-2.67 [-3.94, -1.40]
Su et al.,2019	4.06	2.25	10	59.94	5.41	10	0.0%	-12.92 [-17.47, -8.37]
Tian et al.,2015	45.59	5.33	8	73.42	6.71	8	0.0%	-4.34 [-6.33, -2.35]
**Subtotal (95% CI)**			**16**			**16**	**7.7%**	**-2.66 [-3.68, -1.64]**
Heterogeneity: Tau² = 0.00; Chi² = 0.00, df = 1 (P = 0.98); I² = 0%								
Test for overall effect: Z = 5.10 (P < 0.00001)								
**Total (95% CI)**			**183**			**180**	**100.0%**	**-1.57 [-1.92, -1.22]**
Heterogeneity: Tau² = 0.28; Chi² = 34.76, df = 18 (P = 0.01); I² = 48%								
Test for overall effect: Z = 8.72 (P < 0.00001)								
Test for subgroup differences: Chi² = 7.38, df = 5 (P = 0.19), I² = 32.3%								

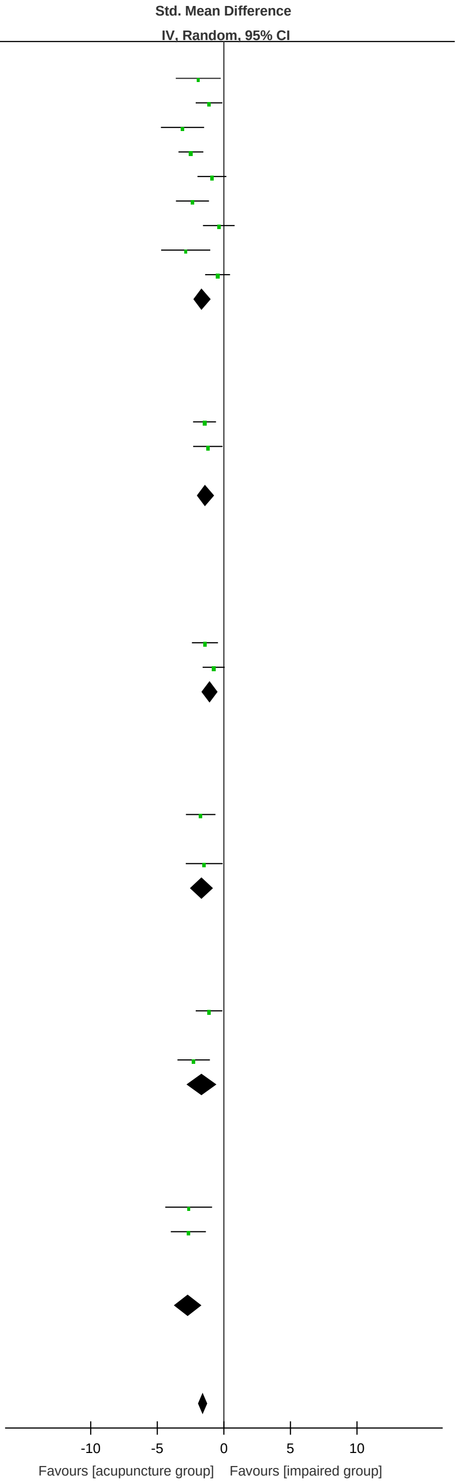

FIGURE 3: Sensitivity analysis of escape latency. Abbreviations: MA (Manual acupuncture); EA (Electroacupuncture). Baihui (GV20) + bilateral Zusanli (ST36), Baihui (GV20) + Shenting (GV24), Baihui (GV20) + Dazhui (GV14), Baihui (GV20) + Dazhui (GV14) + bilateral Shenshu (BL23), Qihai (CV6) + Zhongwan (CV12) + Danzhong (CV17) + bilateral Xuehai (SP10) + bilateral Zusanli (ST36), Other Acupoints (Acupuncture points and methods are different for each study).

Figure 4. Sensitivity analysis of number of crossings

Study or Subgroup	acupuncture group Mean	SD	Total	impaired group Mean	SD	Total	Weight	Std. Mean Difference IV, Random, 95% CI
**1.3.1 CV6 + CV12 + CV17 + SP10 + ST36 (MA)**								
Liu et al.,2006	2.28	1.95	15	1.51	0.81	14	12.8%	0.49 [-0.25, 1.24]
Pan et al.,2021	2.09	1.05	10	1.4	1.05	10	9.4%	0.63 [-0.27, 1.53]
Xu et al.,2022	2	0.85	12	1.17	0.84	12	10.3%	0.95 [0.10, 1.80]
Zhang et al.,2014	2.3	0.54	10	1.62	0.4	10	7.9%	1.37 [0.37, 2.37]
**Subtotal (95% CI)**			**47**			**46**	**40.4%**	**0.80 [0.37, 1.23]**
Heterogeneity: Tau² = 0.00; Chi² = 2.16, df = 3 (P = 0.54); I² = 0%								
Test for overall effect: Z = 3.67 (P = 0.0002)								
**1.3.2 GV20 + GV24 (EA)**								
Feng et al.,2013	2.61	1.31	15	0.61	0.76	15	10.0%	1.82 [0.95, 2.69]
Ma et al.,2022	3.43	1.6	12	3.06	1.89	12	0.0%	0.20 [-0.60, 1.01]
Wang et al.,2021	4.8	0.78	12	3.61	1.15	12	9.8%	1.17 [0.29, 2.05]
**Subtotal (95% CI)**			**27**			**27**	**19.8%**	**1.50 [0.86, 2.13]**
Heterogeneity: Tau² = 0.01; Chi² = 1.06, df = 1 (P = 0.30); I² = 5%								
Test for overall effect: Z = 4.62 (P < 0.00001)								
**1.3.3 GV20 + GV14 (EA)**								
Li et al.,2007	5.75	2.09	12	2.55	1.86	11	8.5%	1.55 [0.60, 2.51]
Wang et al.,2004	5.97	2.13	14	2.31	1.84	13	9.2%	1.78 [0.87, 2.69]
**Subtotal (95% CI)**			**26**			**24**	**17.7%**	**1.67 [1.01, 2.33]**
Heterogeneity: Tau² = 0.00; Chi² = 0.11, df = 1 (P = 0.74); I² = 0%								
Test for overall effect: Z = 4.96 (P < 0.00001)								
**1.3.4 Other Acupoints (MA or EA)**								
Bu et al.,2022	2.65	0.53	6	0.82	0.42	6	0.0%	3.53 [1.47, 5.60]
Guo et al.,2020	8.01	0.57	10	4.61	0.71	10	0.0%	5.06 [3.10, 7.01]
Liu et al.,2017	2.44	2.67	9	1.11	1.88	8	8.2%	0.54 [-0.43, 1.52]
Ma et al.,2020	1.25	0.82	8	0.38	0.48	8	6.7%	1.22 [0.13, 2.32]
Qiu et al.,2022	4.52	1.47	10	2.21	1.09	10	7.2%	1.71 [0.65, 2.77]
Su et al.,2019	7.99	2.36	10	0.94	0.13	10	0.0%	4.04 [2.39, 5.69]
Tian et al.,2015	5.96	0.78	8	2.87	0.75	8	0.0%	3.82 [2.01, 5.63]
**Subtotal (95% CI)**			**27**			**26**	**22.1%**	**1.13 [0.45, 1.81]**
Heterogeneity: Tau² = 0.08; Chi² = 2.59, df = 2 (P = 0.27); I² = 23%								
Test for overall effect: Z = 3.24 (P = 0.001)								
**Total (95% CI)**			**127**			**123**	**100.0%**	**1.17 [0.87, 1.47]**
Heterogeneity: Tau² = 0.04; Chi² = 12.04, df = 10 (P = 0.28); I² = 17%								
Test for overall effect: Z = 7.54 (P < 0.00001)								
Test for subgroup differences: Chi² = 6.06, df = 3 (P = 0.11), I² = 50.5%								

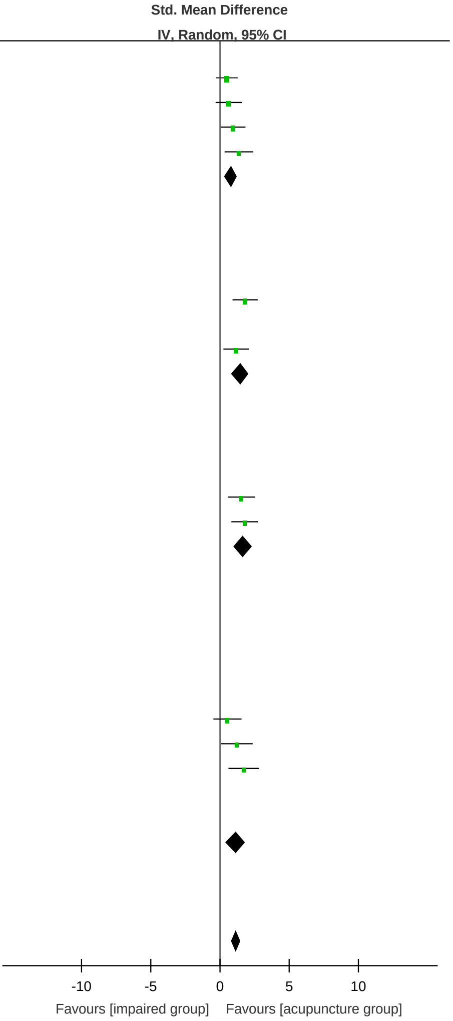

FIGURE 4: Sensitivity analysis of number of crossings. Abbreviations: MA (Manual acupuncture); EA (Electroacupuncture). Qihai (CV6) + Zhongwan (CV12) + Danzhong (CV17) + bilateral Xuehai (SP10) + bilateral Zusanli (ST36), Baihui (GV20) + Shenting (GV24), Baihui (GV20) + Dazhui (GV14), Other Acupoints (Acupuncture points and methods are different for each study).
